# Switching Roles—Exploring Concentration-Dependent Agonistic versus Antagonistic Behavior of Integrin Ligands

**DOI:** 10.1021/acs.jmedchem.4c02111

**Published:** 2025-02-05

**Authors:** Beatrice Stefanie Ludwig, Nils Krautkremer, Stefano Tomassi, Salvatore Di Maro, Francesco Saverio Di Leva, Anke Benge, Markus Nieberler, Horst Kessler, Luciana Marinelli, Susanne Kossatz, Ute Reuning

**Affiliations:** 1 Department of Nuclear Medicine, School of Medicine & Health, Klinikum rechts der Isar, TUM University Hospital, Technical University of Munich, Ismaninger Strasse 22, Munich 81675, Germany; 2 Central Institute for Translational Cancer Research (TranslaTUM), School of Medicine & Health, Klinikum rechts der Isar, TUM University Hospital, Technical University of Munich, Ismaninger Strasse 22, Munich 81675, Germany; 3 Department of Oral and Maxillofacial Surgery, School of Medicine & Health, Klinikum rechts der Isar, TUM University Hospital, Technical University of Munich, Ismaninger Strasse 22, Munich 81675, Germany; 4 UNINA − Department of Pharmacy, University of Naples Federico II, Via Domenico Montesano 49, Naples 80131, Italy; 5 SUN − Department of Environmental, Biological and Pharmaceutical Sciences and Technologies, Università degli Studi della Campania “Luigi Vanvitelli”, Viale Abramo Lincoln, 5, Caserta 81100, Italy; 6 Department of Obstetrics & Gynecology, School of Medicine & Health, Clinical Research Unit, Klinikum rechts der Isar, TUM University Hospital, Technical University of Munich, Ismaninger Strasse 22, Munich 81675, Germany; 7 Department of Chemistry, School of Natural Sciences and Bavarian NMR Center (BNMRZ), Institute for Advanced Study, Technical University Munich, Lichtenbergstrasse 2a, Garching 85748, Germany; 8 Department of Chemistry, School of Natural Sciences, Technical University Munich, Ismaninger Strasse 22, Munich 81675, Germany

## Abstract

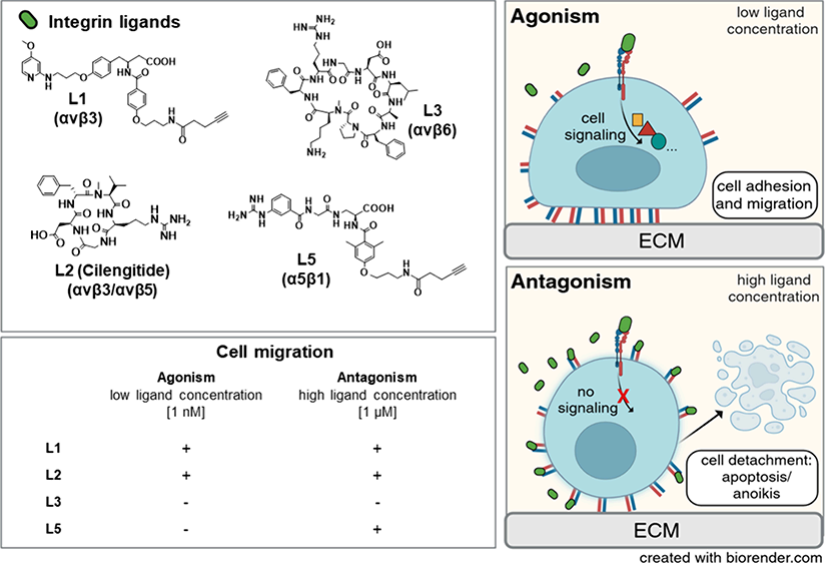

Identification of integrins as cancer targets has stimulated the development of specific inhibitory ligands. However, following cilengitide′s unexpected clinical failure by promoting angiogenesis at low concentrations, pure ligand antagonism was soon scrutinized. We evaluated αvβ3, αvβ6, or α5β1 ligands for concentration-dependent functional switches in respective integrin subtype-overexpressing cancer cells. Cilengitide (L2) or L1 provoked minor transient changes in (p)-FAK and (p)-p44/42^(erk-1/2)^ predominantly at low concentrations and antagonized cell migration at high concentrations, while agonistically accelerating it at low concentrations. L5 (α5β1) showed bell-shaped FAK activation at both concentrations, blocking cell migration at high concentrations only in α5β1+ OV-MZ-6 cells, not acting agonistically. L3 (αvβ6) did not alter signaling upon long exposure but transiently and early activated FAK in αvβ6+ HN cells at both concentrations, with neither antagonistic nor agonistic consequences on cell motility. These data underscore the need for in-depth evaluation of ligand actions to ensure their most promising medical use.

## Introduction

Integrins are heterodimeric transmembrane adhesion receptors, formed by the noncovalent linkage of one out of 18 α- and one out of 8 β-subunits.^1,2^ Upon binding to proteins of the extracellular matrix (ECM), they are capable of bidirectional signaling across cell membranes, thereby mediating biological processes, such as cell adhesion, migration/invasion, proliferation, and survival.^2−7^ Under normal quiescent conditions, integrins are in a low-affinity binding state. During their activation, via *inside-out* signaling, conformational changes occur involving the dissociation of integrin transmembrane domains (TMDs) and cytoplasmic regions, followed by the binding of cytoplasmic talin and kindlin. This allows the homooligomerization to α-dimers and ß-trimers, which cluster with other integrin subtypes and receptor proteins on the cell membrane. In focal adhesions (FAs), integrins bind with high affinity to ECM ligands in a multivalent manner. This triggers *outside-in* signaling via the phosphorylation and thus activation of important downstream signaling molecules, such as the focal adhesion kinase (FAK) and the mitogen-activated protein kinases (MAPK) p44/42^(erk-1/2)^. This finally leads to altered gene expression and cell biological events, crucial for rapid cell responses to microenvironmental demands.^8−18^

Due to their key cellular functions, integrins are also involved in pathophysiological events, including tumor growth, invasion, metastasis, and angiogenesis. Eight integrin subtypes that recognize the tripeptide motif Arg-Gly-Asp (RGD) within ECM proteins are overexpressed in certain cancer entities, identifying them as valuable and accessible targets for cancer therapy and diagnostic imaging.^4−7,19^ Consequently, RGD-based synthetic ligands with high integrin subtype selectivity and affinity were developed to interfere with endothelial and cancer cell seeding onto the ECM, thereby driving these *per se* anchorage-dependent cells into apoptosis (anoikis) via the “integrin-mediated cell death” (IMD).^7,20−27^ Following the observation in clinical trials that the αvβ3-targeting ligand cilengitide (L2), originally designed to target αvβ3-mediated adhesion of neoangiogenic endothelial cells, failed to induce antiangiogenic effects, the paradigm of purely antagonistic ligand performance was soon questioned. In fact, a low concentration of cilengitide (L2) turned out to act as an agonist, resulting in the promotion of tumor angiogenesis and vascular stabilization by promoting vascular endothelial growth factor (VEGF)-mediated angiogenesis via enhanced VEGF receptor 2 expression and signaling and finally endothelial cell migration. Moreover, increased αvβ3 recruitment to FA was noticed, enhancing integrin affinity and signaling and thus tumor cell proliferation and enhanced angiogenesis.^28−36^ Thus, the use of cilengitide (L2) was soon repurposed by exploiting its vascular stabilization effects at a minor concentration for the improvement of tumor blood flow and perfusion through the per se rather immature and leaky angiogenic tumor vasculature, thereby enhancing the delivery of cancer therapeutics.^28,32,37,38^ Meanwhile, various ligands targeting the RGD-binding integrins αvβ3, αvβ6, or α5β1 have been developed to selectively target tumors which overexpress the respective integrin subtype.^5,7,21,39−45^ However, it still remains questionable whether the concentration-dependent functional switch of cilengitide (L2) may also be relevant for other integrin ligands.

In principle, ligands may bind not only to the initial heterodimeric integrin state but also to binding sites within FAs. Agonistic performance is suggested to be evoked by ligand-induced conformational changes within the integrin molecule leading to its activation into a high-affinity and signaling-competent receptor.^14,15,32,36,46,47^ Indeed, it was shown that cilengitide (L2) primed αvβ3 to transform from a compact-closed to an extended-open conformation in the absence of any other exogenously activating stimulus *in vitro.*(28,30) On the opposite, preventing TMD dissociation is thought to result in pure antagonism, without the induction of integrin signaling. To this end, the characterization of ligand actions on integrins and the cellular effects arising thereof are of special importance, especially since even upon high-dose systemic treatments, low ligand concentrations at the tumor site are unavoidable due to their rapid pharmacokinetics and short half-lives.^45,48^ Thus, in cancer cells overexpressing either αvβ3, αvβ6, or α5β1, we aimed to decipher antagonistic from agonistic behavior of a panel of previously well-characterized RGD-based ligands^49^ by monitoring their internalization and concentration-dependent capacity to trigger integrin signaling. We determined the activation/phosphorylation state of the integrin downstream signaling molecules FAK and MAPK p44/42^(erk-1/2)^ and, as biological readout, the integrin-dependent cell migratory and adhesive activity. This approach is thought to shed light on the biological performance of ligands to identify the most suitable compounds, e.g., for anticancer therapy, tumor imaging, or possible vascular stabilization prior to their intended medical use.

## Results

### Synthesis of Integrin Ligands Targeting αvβ3, αvβ6, or α5β1

**Figure 1:**
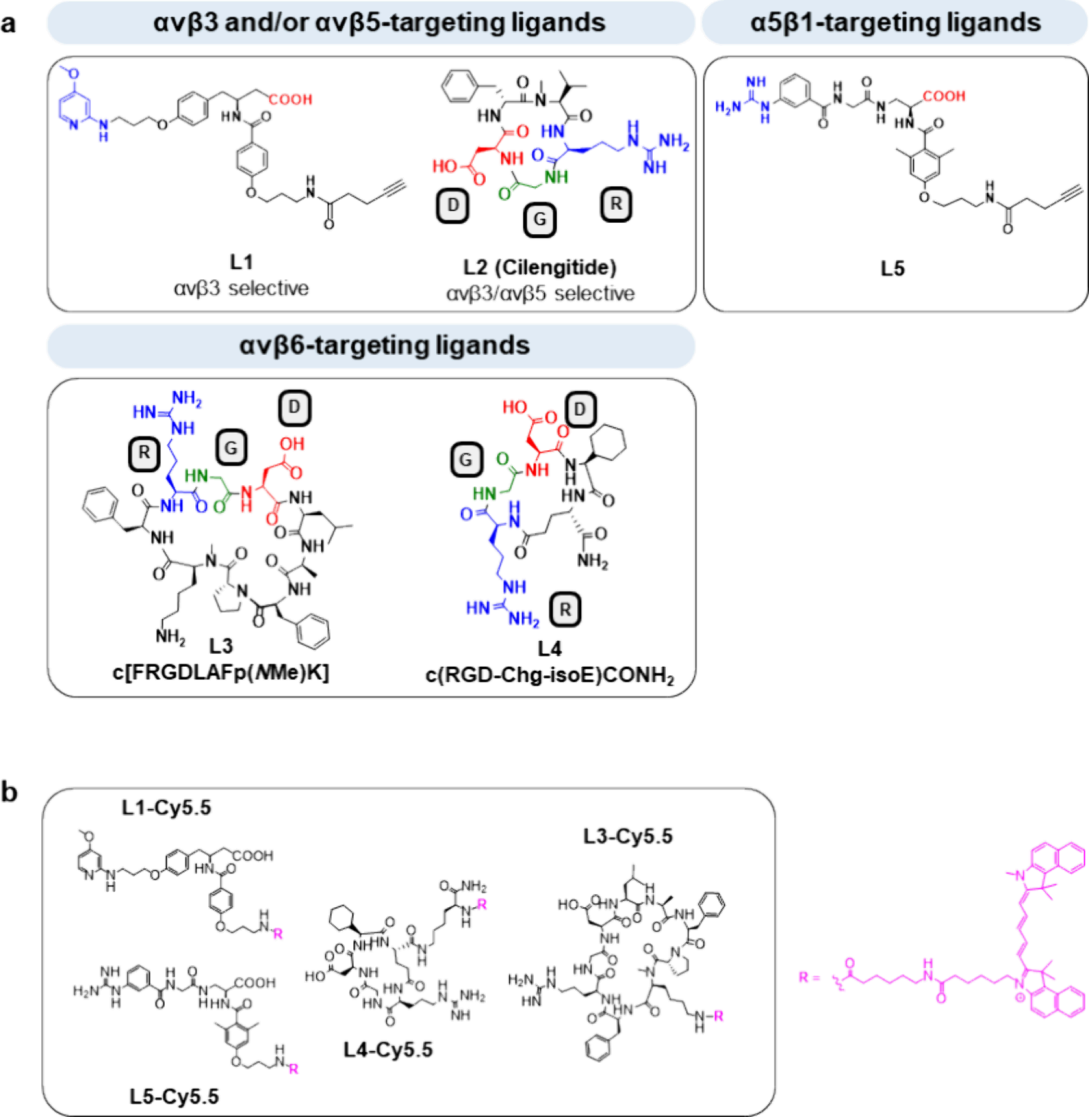
Chemical structures of the integrin ligands (a) and their Cy5.5-labeled counterparts (b). The integrin-binding motif RGD is shown in blue–green–red. In pink, the Cy5.5 motif.

We synthesized five highly affine and selective compounds based on the previously published RGD-based ligands: for αvβ3, the peptidomimetic L1 and cilengitide (L2),^39−41^ for αvβ6, the peptide ligands L3^42^ and L4,^43^ and for α5β1, the peptidomimetic compound L5^41,44^ (Figure 1). L1 and L3 were functionalized with a nonamine group, containing a functional group (alkine moiety) in order to prevent unspecific binding. Cilengitide (L2) is still considered as a reference compound for integrin binding characteristics of new integrin ligands and also served here as an internal positive control for agonistic (at low concentrations) and antagonistic (at high concentrations) αvβ3/α5β1-dependent effects. For monitoring time-dependent cellular ligand uptake, compounds L1, L3, L4, and L5 were labeled with the fluorescence dye Cy5.5 via an aminohexanoic acid linker. Yields of all generated compounds ranged between 6 and 28% with purities greater than 96% by HPLC (see Scheme S1 in the Supporting Information).

**Figure 2:**
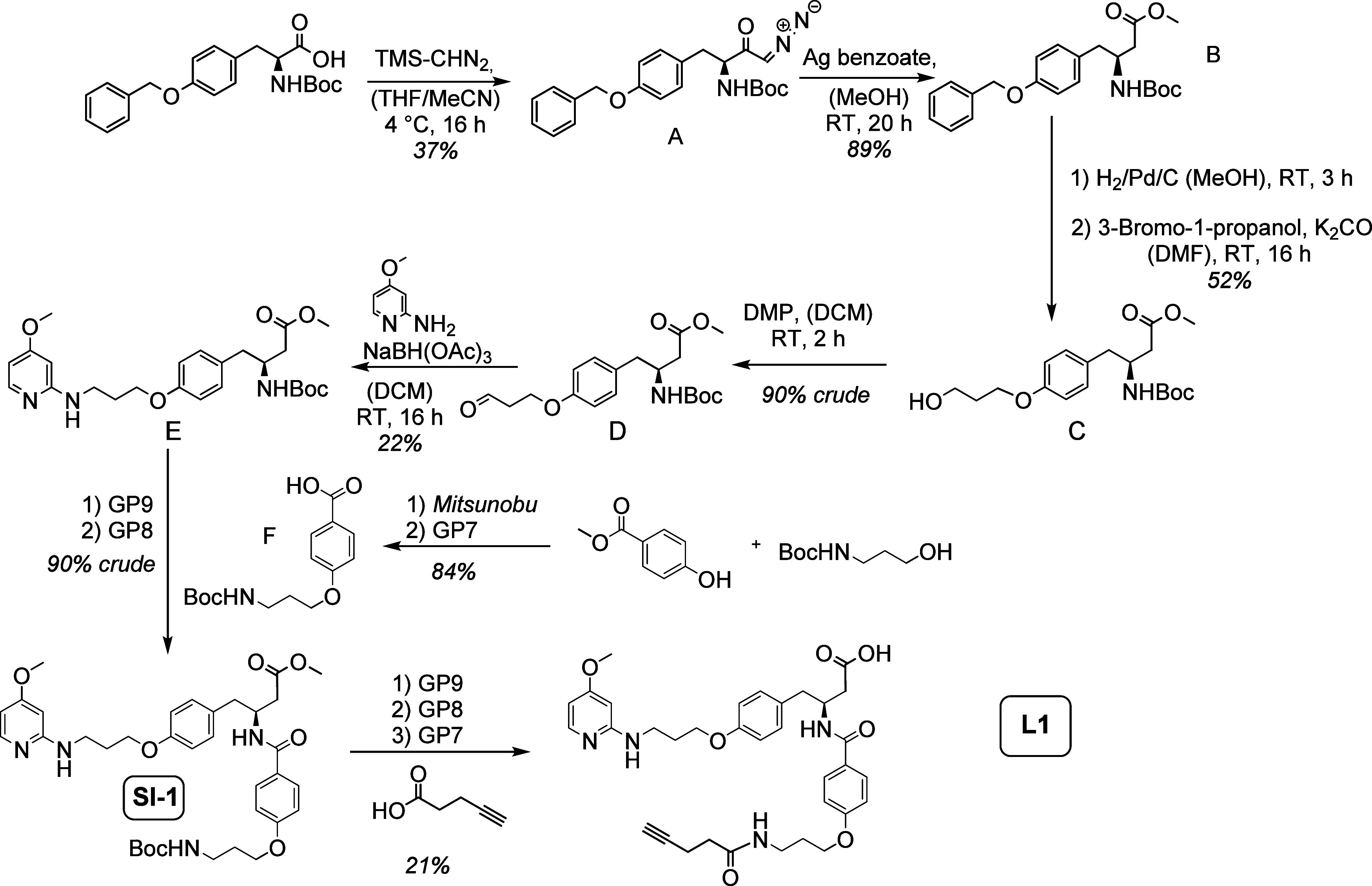
Reaction steps for the synthesis of L1.^40,41,44,49^ Prepared from (*S*)-methyl-3-(*tert*-butoxycarbonylamino)-4-(4-(3-(4-methoxypyridin-2-ylamino)propoxy)phenyl)butanoate^41^ (20 mg, 42.3 μmol, 1.00 equiv) according to the following reaction sequence: After Boc deprotection (GP9), 4-(3-((*tert*-butoxycarbonyl)amino)propoxy)benzoic acid (15 mg, 50.7 μmol, 1.20 equiv) was coupled according to GP8. The resulting crude product SI-1 was again Boc-deprotected (GP9) and coupled to 4-pentynoic acid (GP8; 4.45 mg, 45.3 μmol, 1.20 equiv). Saponification according to GP7 and semipreparative RP-HPLC purification (20–60%, buffer B, 15 min) resulted in L1 (5.87 mg, 9.51 μmol, 21%) as a yellowish solid.

The synthesis of peptidomimetic L1 was accomplished in accordance with a simplified method from the literature (Figure 2). In the first step, β-Tyr C was generated via an *Arndt–Eistert* homologation. Therefore, trimethylsilyl diazomethane was reacted with the mixed anhydride of Boc-Tyr(Bzl)-OH and ethyl chloroformate, resulting in the isolable diazoketone A in a yield of 37%.^50^

Subsequent addition of the Ag+ salt silver benzoate initiated the *Wolff* rearrangement, the second step of the *Arndt–Eistert* homologation, leading to methyl ester B in a yield of 89%. After the hydrogenation of ester B with palladium on carbon and nucleophilic substitution with 3-bromopropanole, alcohol C was obtained in 52% yield. In the next step, alcohol C was oxidized to aldehyde D by addition of *Dess*–*Martin* periodinane. As the aldehyde was unstable, it was directly processed without further purification steps in a reductive amination. Here, imine formation with 2-amino-4-methoxypyridine occurred *in situ* in the presence of MgSO_4_. Subsequent reduction by addition of sodium triacetoxyborohydride resulted in the desired peptidomimetic ligand scaffold E, obtained in 22% yield as a yellowish oil. Starting from E, Boc deprotection in acidic milieu and coupling of F resulted in SI-1 in 90% yield. After deprotection of acid-labile Boc, coupling of 4-pentynoic acid, and global deprotection (trifluoroacetic acid (TFA) for acid labile groups, LiOH for base labile groups), the integrin ligand L1 was acquired in 21% yield.

**Figure 3:**
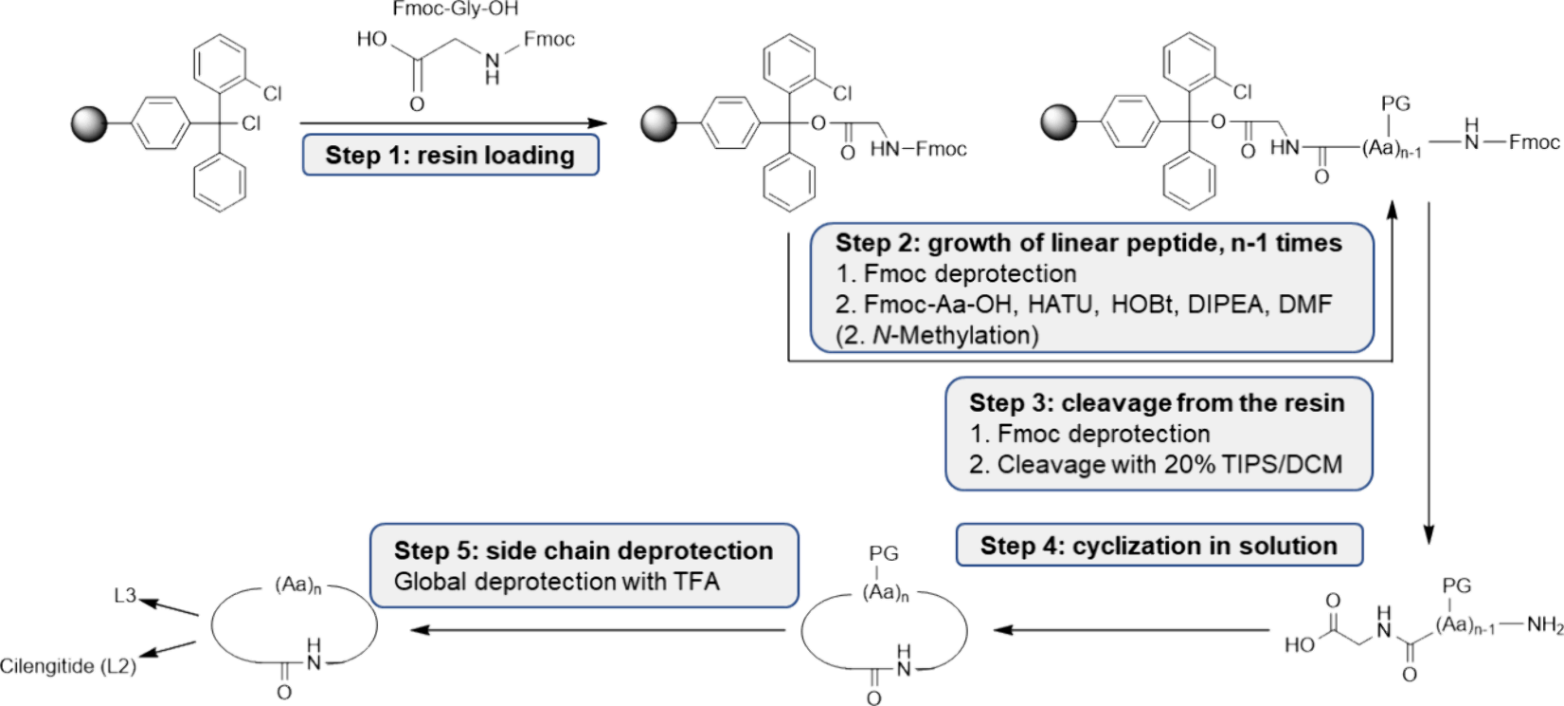
Fmoc-based synthesis of cyclic peptides on a solid support (used for the synthesis of L2 and L3). Step 1: the resin was loaded with the first amino acid, Fmoc-Gly-OH. Step 2: chain elongation was performed using the coupling reagents HATU/HOBt and DIPEA as base after Fmoc deprotection (20% (*v*/*v*) piperidine in DMF). Also, after Fmoc deprotection, *N*-methylation was performed. Steps 3 and 4: After the final Fmoc deprotection, the linear peptide was cleaved from the resin with 20% (*v*/*v*) TIPS/DCM under maintenance of the side-chain protection groups. Then, cyclization in solution was performed. Step 5: Global side-chain deprotection with TFA.

**Figure 4:**
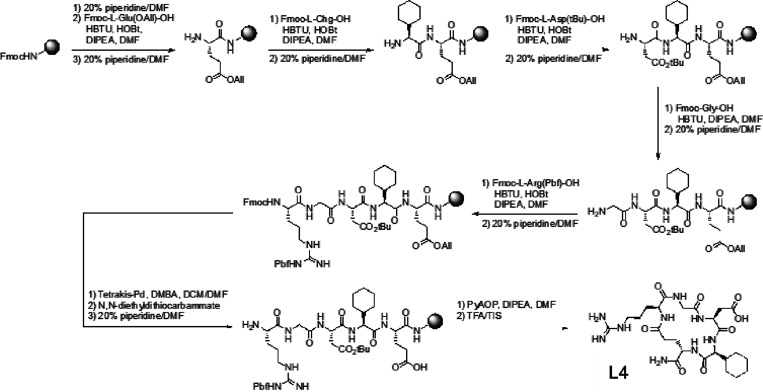
Overview of the reaction steps for the synthesis of L4. The cyclic peptide was synthesized on solid support by a canonical Fmoc/*t*Bu approach in accordance to the protocol appeared in literature.^43,49^

The cyclic ανβ3- and ανβ6-targeting integrin ligands L2, L3, and L4 were synthesized according to a standard Fmoc-protocol^51,52^ on the solid phase applying a literature-known procedure (Figure 3).^42,53−56^ In a first step, the solid support, 2-CTC resin, was loaded with Fmoc-Gly-OH. Fmoc-Aa-OH was preactivated with HATU/HOBt in DIPEA/DMF for a few minutes before resin addition. As free side-chain groups lead to cross-reactions during the coupling step, all amino acids within this work were used with orthogonally protected side chains. After 1 h of coupling, a quantitative turnover was achieved (analyzed via HPLC-MS). For *N*-methylation, the Fmoc group was first deprotected with piperidine following Ns-protection (*N*-monosubstituted nitrobenzenesulfonamide generation). To finally obtain the desired *N*-methylated secondary amine, treatment with thiolate nucleophiles such as mercaptoethanol led to rapid Ns deprotection within 5 min via the generation of a *Meisenheimer* complex.^57^ After cleavage of the linear peptides from the resin under maintenance of the side-chain protection groups with TIPS/DCM, and cyclization in solution, the peptides were globally deprotected with TFA (acid labile groups) for receiving integrin ligands L2 (cilengitide) and L3. L4 was synthesized in a similar way (Figure 4).

Subsequently, chain elongation was performed by Fmoc deprotection with piperidine and coupling of the next Fmoc-protected amino acid (Fmoc-Aa-OH) using DIPEA as the base as well as HATU/HOBt as coupling agents. Since the coupling agents act as auxiliary nucleophiles, the carboxylic acid moiety of Fmoc-Aa-OH is activated, thus making it accessible for peptide coupling.

**Figure 5:**
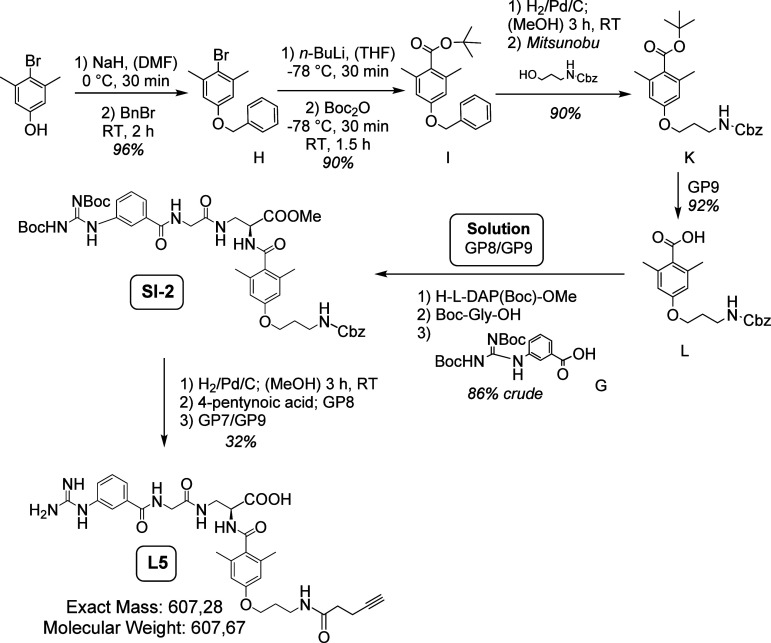
Overview of the reaction steps for the synthesis of L5.^40,41,49^ Following the Boc strategy, the ligand was synthesized in-solution. L5 was prepared from *^t^*Bu-deprotected (GP9) benzyl 4-(4-(*tert*-butoxycarbonyl)-3,5-dimethylphenoxy)butylcarbamate^41^ (20 mg, 56 μmol, 1.05 equiv), in compliance with the following reaction sequence: coupling of H-l-Dap(Boc)-OMe (GP8), Boc deprotection (GP9), coupling of Boc-Gly-OH (GP8), Boc deprotection (GP9), coupling of (*Z*)-3-(2,3-Bis(*tert*-butoxycarbonyl)guanidino)benzoic acid^49^ (GP8), Cbz deprotection with H_2_/Pd/C in MeOH for 3 h, coupling of 4-pentynoic acid (GP9), and global deprotection according GP7 and GP9. Purification via semipreparative RP-HPLC (20–60% buffer B, 15 min) led to compound L5 (10.3 mg, 17 μmol, 32%) as white solid.

With regard to L5, the building blocks G, H, I, K, and L were synthesized in a multistep reaction sequence in solution according to a literature procedure (Figure 5).^58^ In this context, 4-bromo-3,5-dimethylphenol was converted to benzyl-protected derivative H via a *Williamson* ether synthesis. The yield of H was 96% after column chromatography. During the *Williamson* ether synthesis, the nucleophilic phenol attacked the electrophilic reagent benzyl bromide, generating NaBr as a white, insoluble side product. In the subsequent step, a *t*Bu-protected acid moiety was introduced by the attack of Boc_2_O at the nucleophilic carbon center that was formed after *n*-BuLi treatment of H (bromine–lithium exchange). In that case, the desired product I as a white crystalline solid with a yield of 90% was formed together with CO_2_ and *t*butanol. After palladium-catalyzed hydrogenation of *t*Bu-protected acid I, the resulting phenol was reacted with benzyl-*N*-(3-hydroxypropylcarbamate) in a Mitsunobu reaction to give compound L in an overall yield of 90%. *t*Bu-deprotected L was conjugated to H-l-Dap(Boc)-Ome, Boc-Gly-OH, and G employing the coupling reagents TBTU and HOBt. The final α5β1-targeting ligand L5 was obtained in a yield of 32% after coupling of 4-pentynoic acid and global deprotection in a yield of 32%.

**Figure 6:**
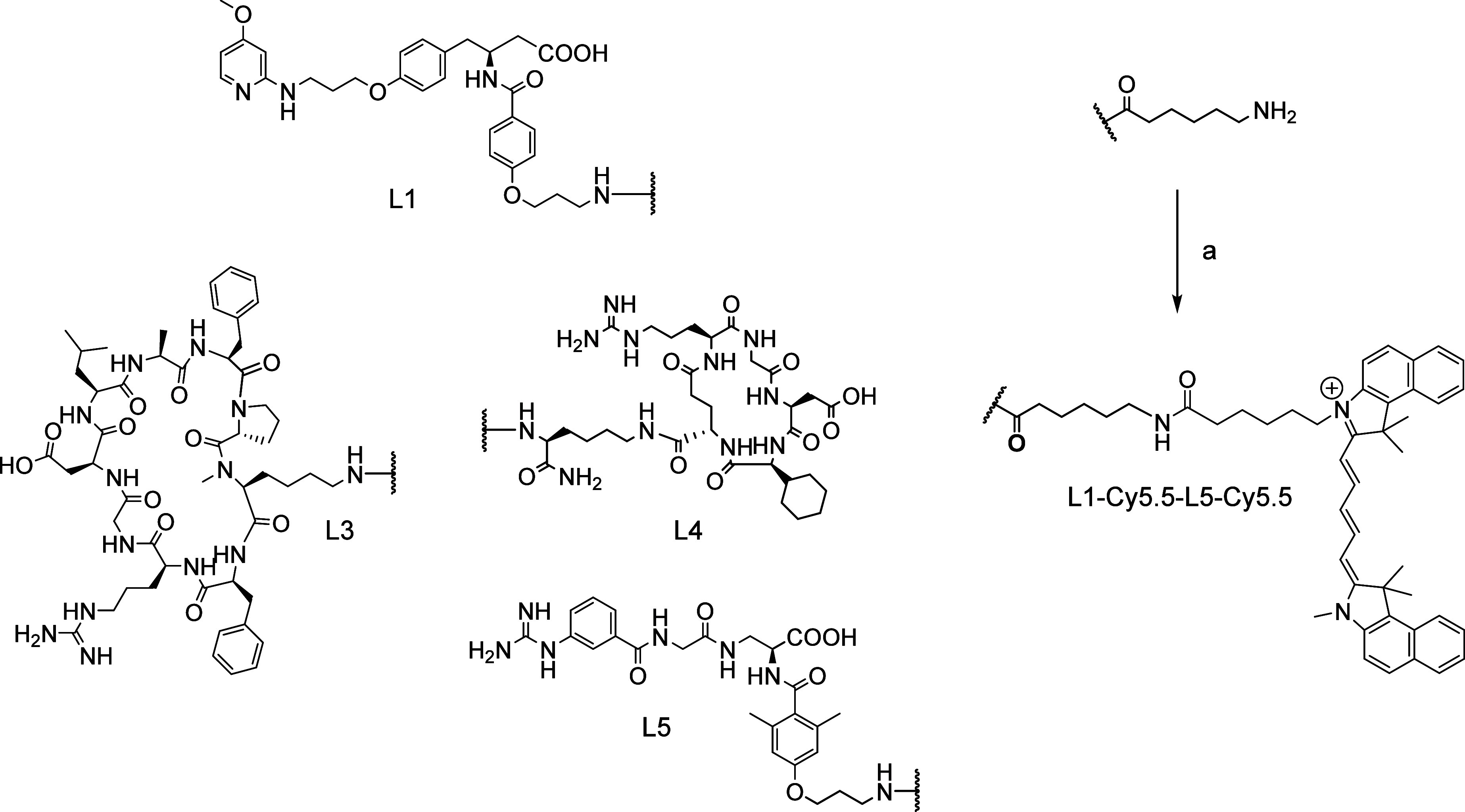
Schematic synthesis of Cy5.5-conjugated integrin ligands L1-Cy5.5 to L5-Cy5.5. a: Cy5.5-NHS ester, DIPEA, DMF.

In order to produce Cy5.5-conjugated integrin ligands (L1-Cy5.5 to L5-Cy5.5) for intracellular tracking, an Ahx-spacer unit was inserted between Cy5.5 and the integrin ligand (Figure 6). Due to the bulky Cy5.5-moiety, the Ahx-linker was used to preserve optimal binding of the ligand into the integrin receptor cavity. For conjugation to the 6-Ahx extended ligands, Cy5.5-NHS ester was added to a solution of the integrin ligand in DIPEA/DMF. After 2–3 h, a completion of the reaction was observed via LC-MS. The final Cy5.5-conjugates were obtained as blue, hygroscopic solids in yields ranging from 48 to 86% after RP-HPLC purification.

### Biological Evaluation of the Integrin Ligands

Prior to the biological evaluation of the ligands, their specificity and subtype-selective affinity was analyzed by an ELISA (enzyme-linked immune sorbent assay)-type solid-phase ligand binding assay^59^ by measuring the competitive binding of soluble purified integrins in the presence of the respective ligands to plate-immobilized purified ECM proteins, as described in Experimental Section: vitronectin (for αvβ3-directed ligands), fibronectin (for α5β1-directed ligands), or the latency-associated peptide (LAP) of transforming growth factor-β (TGF-β) (for αvβ6-directed ligands). Then, we determined whether Cy5.5 conjugation of ligands led to altered ligand binding affinities and integrin subtype selectivity compared to the unlabeled compounds. The obtained IC_50_ values for all ligands and their respective Cy5.5-labeled counterparts are given in Table 1. In the following, we describe in detail the alterations detected.

**Table 1:** Binding Profiles of L1–L5 and the Corresponding Cy5.5-Conjugates^a^^59^

compound	IC_50_ αvβ3 [nM]^b^	IC_50_ α5β1 [nM]^b^	IC_50_ αvβ6 [nM]^b^
α***v***β***3-selective ligands***			
L1	**1.10 ± 0.27**	73.4 ± 16.6	152 ± 60.8
L1-Cy5.5	**1.02 ± 0.02**	>1000	>1000
α***v***β***6-selective ligands***			
L3^c^	>1000	73 ± 6	0.28 ± 0.019
L3-Cy5.5	196 ± 78.9	>1000	**3.64 ± 4.11**
L4^c^	364 ± 96	105 ± 11	**1.3 ± 0.2**
L4-Cy5.5	15.0 ± 5.79	>1000	**7.63 ± 0.83**
α***5***β***1-selective ligands***			
L5	>1000	1.24 ± 0.04	>1000
L5-Cy5.5	>1000	**4.69 ± 1.00**	154 ± 136
***Internal reference ligands***			
cilengitide (L2)^c^	**0.61 ± 0.06**	14.9 ± 3.1	>1000
RTDLDSLRT^c^	>1000	>1000	**29.5 ± 4.5**

a The selectivity profile of the ligands for RGD-recognizing integrins was determined by a competitive ELISA-type solid-phase binding assay using coated ECM proteins and soluble integrins.

b The IC_50_ values were obtained using a sigmoidal fit to 16 data points, received from two serial dilution and by referencing to the affinity of the internal standards, cilengitide (L2), or RTDLDSLRT.

c IC_50_ values of L3, L4,^42^ cilengitide (L2), and RTDLDSLRT have previously been published.^59^

### Integrin αvβ3 Ligands L1 and Cilengitide (L2)

The affinity of L1-Cy5 (IC_50_ value L1-Cy5.5: 1.01 nM) for αvβ3 was almost comparable to that of L1 (IC_50_ value: 1.10 nM), whereas an increased selectivity for L1-Cy5.5 toward α5β1 (IC_50_ value L1-Cy5.5: > 1000 nM, IC_50_ value L1: 73.4 nM) and toward αvβ6 (IC_50_ value L1-Cy5.5: >1000 nM, IC_50_ value L1: 152 nM) was noticed. Cilengitide (L2) bound αvβ3 (IC_50_ value: 0.61 nM) and α5β1 (IC_50_ value: 14.9 nM) with a high affinity.

### Integrin αvβ6 Ligands L3 and L4

L3-Cy5.5 showed a drop of affinity toward αvβ6 (IC_50_ value: 3.64 nM) compared to L3 (IC_50_ value: 0.28 nM) but a significantly increased affinity toward αvβ3 (IC_50_ value: 196 nM) compared to L3 (IC_50_ value >1000 nM). L4-Cy5.5 displayed a slightly lower affinity toward αvβ6 (IC_50_ value: 7.63 nM) compared to L4 (IC_50_ value: 1.3 nM) and a markedly increased affinity toward αvβ3 (IC_50_ value: 15 nM) compared to L4 (IC_50_ value: 364 nM). Both L3-Cy5.5 and L4-Cy5.5 showed a significantly decreased affinity toward α5β1 (IC_50_ value >1000 nM) compared to L3 (IC_50_ value: 73 nM) or L4 (IC_50_ value: 105 nM).

### Integrin α5β1 Ligand L5

L5 and L5-Cy5.5 showed comparable affinities toward α5β1 with 1.2 and 4.6 nM, respectively, and comparably low affinities toward αvβ3 (IC_50_ value >1000 nM). L5-Cy5.5 displayed a decreased selectivity toward αvβ6 (IC_50_ value: 154 nM) compared to L5 (IC_50_ value >1000 nM).

Taken together, all ligands demonstrated IC_50_ values in the low nM range toward their respective target integrin subtype with several orders of magnitude higher values to the nontargeted integrins. In the case of L1, Cy5.5-conjugation even increased the selectivity by decreasing the affinity toward the nontarget integrins. For the other ligands, Cy5.5-conjugation led to a partial increase or decrease in selectivity toward the nontargeted integrins. The affinity toward the target integrin was not substantially altered upon Cy5.5-conjugation.

### Cellular Uptake of Cy5.5-Labeled Integrin Ligands

For intracellular drug delivery, integrin ligands designed as drug-conjugated vehicles have to be easily and selectively internalized by tumor cells (over)expressing the respective targeted integrin subtype. To this end, we performed cellular uptake studies with M21 cells, harboring elevated αvβ3 expression levels, HN cells, displaying high αvβ6 expression, and OV-MZ-6 and RKO cells, exhibiting a high α5β1 content. It should be noted here that HN, OV-MZ-6, and RKO cells also express low αvβ3 expression levels. As a control for selectivity, we incubated the ligands on tumor cell types that do not or only very weakly express the respective targeted integrin subtype.

**Figure 7:**
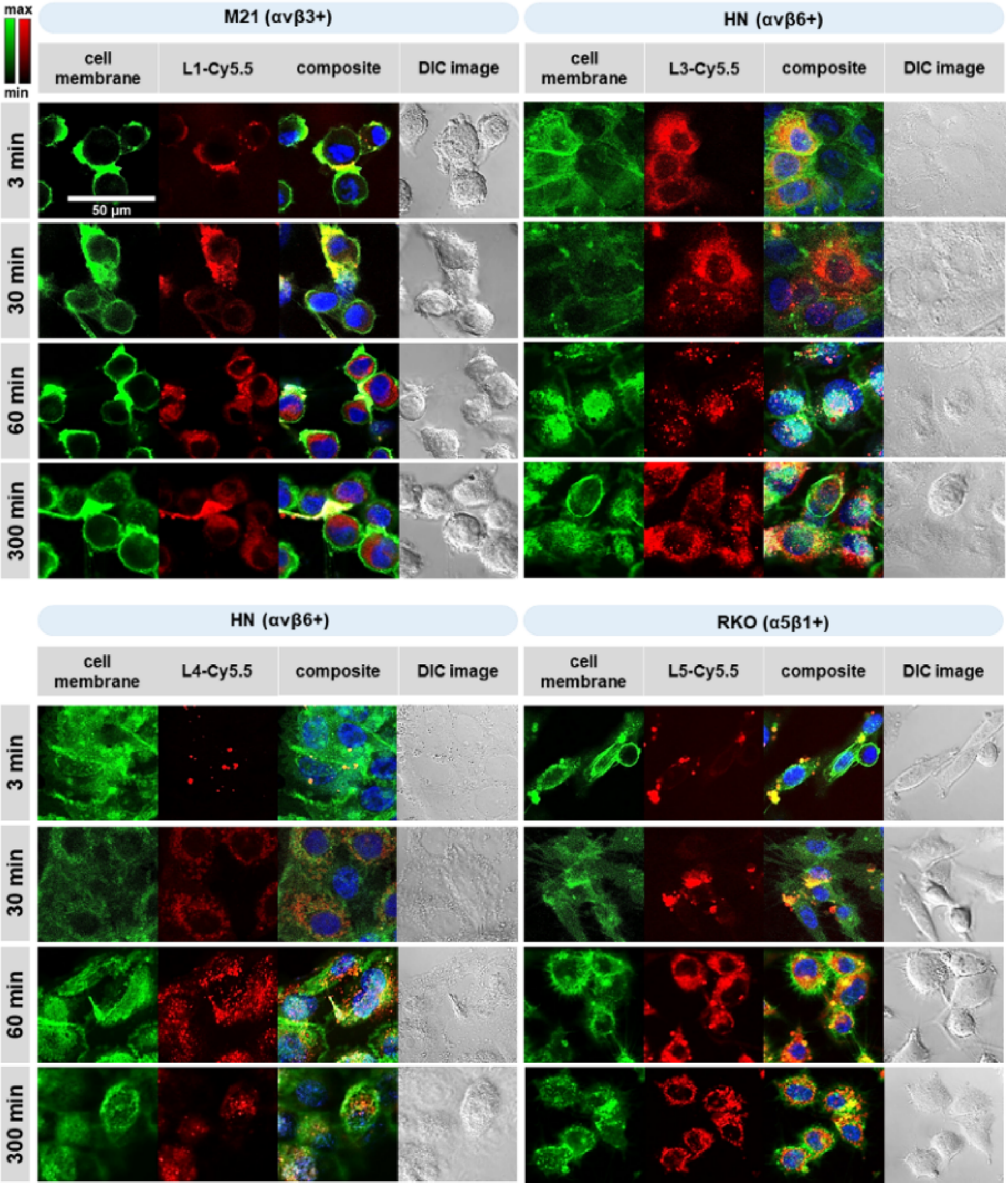
Cellular uptake of Cy5.5-labeled integrin ligands. M21 (L1-Cy5.5, αvβ3+), HN (L3-Cy5.5 and L4-Cy5.5, αvβ6+), and RKO cells (L5-Cy5.5, α5β1+) were treated with 1 μM of the respective integrin ligand (red signal) for 3, 30, 60, or 300 min. Nuclei were costained with Hoechst 33342 (blue signal) and cell membranes with CellMask Green (green signal). Merge of stains (yellow signal) indicated colocalization of ligands at the cell membrane. Scale bar: 50 μm.

Time-dependent cell incubation with the Cy5.5-conjugated ligands led to the following results: already within 3 min, all ligands were associated with cell membranes, whereas exclusively L3-Cy5.5 was already intracellularly visible within HN cells (Figure 7). At 30 min, all ligands were still mostly localized at cell membranes. Within 60 min, all ligands were intracellularly localized. L3-Cy5.5 or L4-Cy5.5 did not bind to αvβ6-OV-MZ-6 cells and also only minor intracellular traces of L1-Cy5.5 were visible. Ligand uptake and intracellular localization persisted up to 300 min and occurred integrin subtype-specific. Since L3 showed better cellular uptake than L4, for all further analyses, only L3 was taken to target αvβ6. Integrin-specific cellular uptake of Cy5.5-labeled integrin ligands was proven by comparing their internalization within 60 or 300 min between cells overexpressing the respective targeted integrin and those with low or lacking expression. This clearly showed the dependence of ligand internalization on an elevated density of the respective target integrin on cellular membranes (see Scheme S2 in the Supporting Information).

### Analysis of Integrin-Mediated Cell Signaling upon Ligand Treatment

**Figure 8:**
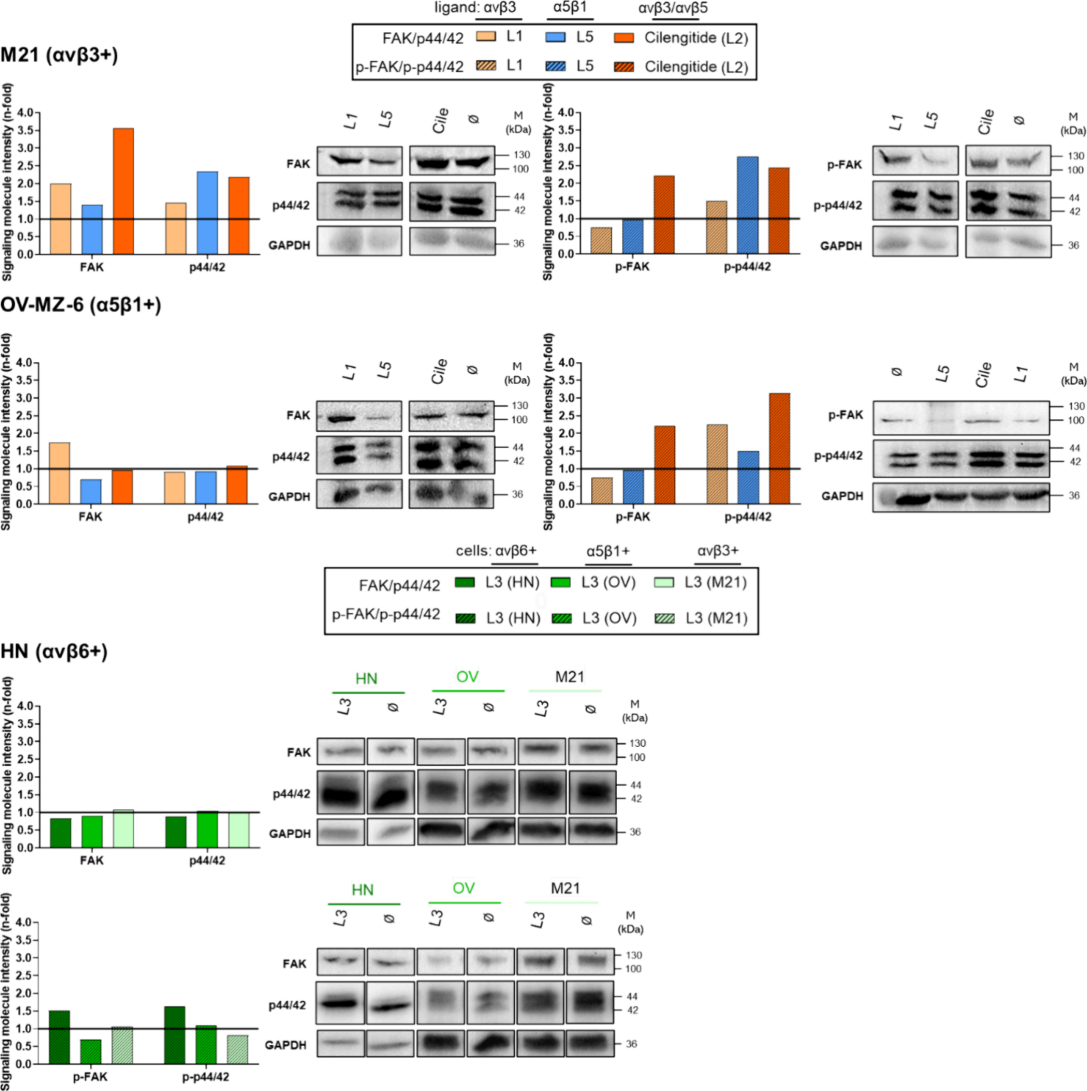
Effects of ligands on cellular expression and activation of integrin-related signaling molecules FAK and p44/42^(erk-1/2)^. Integrin αvβ3+ M21 cells, α5β1+ OV-MZ-6, or αvβ6+ HN cells were incubated with 1 μM of L1 (αvβ3), cilengitide (L2; αvβ3/αvβ5), L3 (αvβ6), or L5 (α5β1) for 24 h, and the cellular expression and activation levels of FAK and p44/42^(erk-1/2)^ were detected by Western blot analysis. Signal intensities for FAK and p-FAK, respectively, were evaluated by use of the Bio-Rad Imager Gel Doc XR+ and a ChemiDoc XRS+ Systems and the software Image Lab. Shown are typical representative Western blot images and the corresponding histograms depicting GAPDH-normalized signal intensities as *n*-fold by setting the values obtained for untreated cells to “1” (drawn as black line). For each cell line, cutouts of identical gels are depicted.

Since agonistic ligand performance requires integrin activation and signaling, by Western blot analysis, we studied whether the different ligands alter the expression levels and activation states of FAK and p44/42^(erk-1/2)^. For this, the respective target integrin-expressing tumor cell types were incubated with the corresponding subtype-selective ligand at a concentration of 1 μM for 24 h (Figure 8). As controls for integrin selectivity of ligands served tumor cells, which do not display or lack significant levels of the respective targeted integrin subtype. Signal intensities of FAK and p44/42^(erk-1/2)^ expression and activation (p-FAK and p-p44/42^(erk-1/2)^) on Western blots were evaluated by densitometry. Data are depicted as GAPDH-normalized fluorescence signal intensities as *n*-fold, by setting values obtained for the respective untreated cells to “1”. The effects may be summarized as follows, when defining alterations in signal intensities above 1.5-fold as the cutoff: cilengitide (L2; αvβ3) provoked an approximately 3.6-fold increased FAK and an approximately 2.2-fold elevated p-FAK expression in αvβ3+ M21 cells. MAPK p44/42^(erk-1/2)^ expression was approximately 2.2-fold augmented, p44/42^(erk-1/2)^ by approximately 2.5-fold. It also raised activation of p-FAK and p-p44/42^(erk-1/2)^ by approximately 2.2-fold and 3.1-fold, respectively, in α5β1+ OV-MZ-6 cells. Also, L1 (αvβ3) led to doubled FAK but slightly reduced p-FAK expression. Additionally, it raised FAK (approximately 1.7-fold) and p-p44/42^(erk-1/2)^ (approximately 2.4-fold) in α5β1+ OV-MZ-6 cells. L3 was not effective in modulating signaling molecules in either cell type. L5 (α5β1) did not modulate (p-)FAK and (p-)p-44/42^(erk-1/2)^ in OV-MZ-6 cells, while it unexpectedly increased (p-)p-44/42^(erk-1/2)^ in M21 cells, possibly due to the presence of small amounts of this integrin in this cancer cell type (see Schemes S3 and S4 in the Supporting Information) (Figure 8).

### Time- and Concentration-Dependent Effects of Integrin Ligands on FAK Expression and Activation

**Figure 9:**
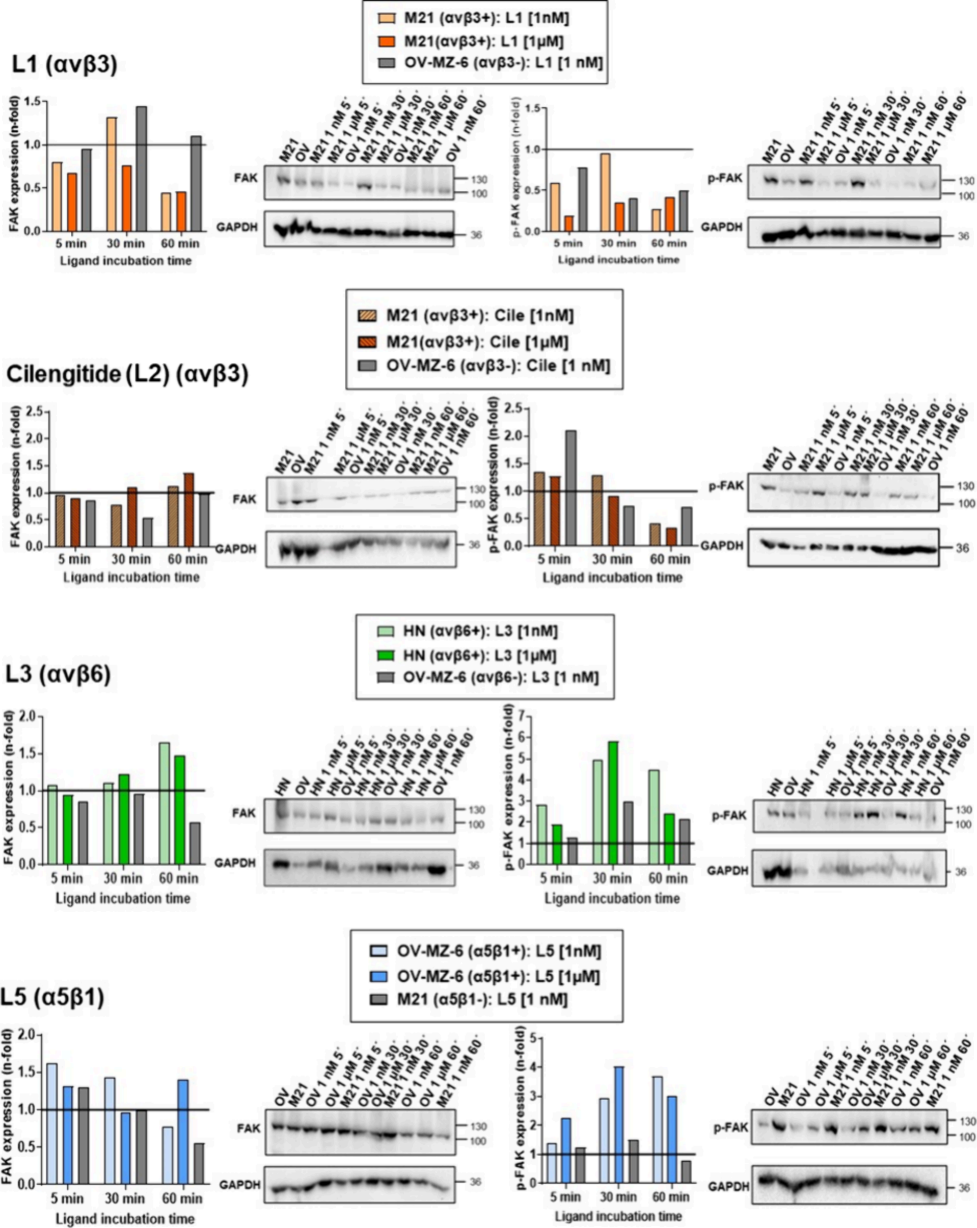
Time- and concentration-dependent effects of integrin ligands L1, L3, L5, or cilengitide (L2) on FAK expression and activation. Integrin αvβ3+ M21, αvβ6+ HN cells, or α5β1+ OV-MZ-6 cells were incubated with 1 μM or 1 nM of L1 (αvβ3), cilengitide (L2; αvβ3/αvβ5), L3 (αvβ6), or L5 (α5β1) for 5, 30, or 60 min. Cellular expression and activation levels of FAK were detected by Western blot analysis. Typical Western blot images are depicted together with the corresponding densitometrically evaluated and GAPDH-normalized signal intensities as described.

In order to capture early and transient changes in signaling activation following ligand treatment, we next reduced the observation periods to 5, 30, or 60 min and assessed FAK expression and activation. Based on the reported concentration-dependent functional switch of cilengitide (L2) from an antagonist to an agonist,^32^ we also here incubated the ligands either at low (1 nM: close to the IC_50_ value of ligands L1–L5) or at high (1 μM) concentration. Depicted are typical Western blot images and the corresponding densitometrical evaluations of GAPDH-normalized signal intensities as *n*-fold, by setting that of the respective untreated cell type to “1” (Figure 9).

#### Integrin αvβ3-Targeting L1

L1 at 1 nM led to a transient and slight increase of FAK expression in αvβ3+ M21 cells, whereas at 1 μM, it remained below that in untreated cells. p-FAK expression at 1 nM was always prominently higher than that at 1 μM, even not exceeding levels in untreated cells. L1 also led to a transient slight increase of FAK and an approximately twofold raised p-FAK in OV-MZ-6 cells (low αvβ3), which continuously decreased within 60 min.

#### Integrin αvβ3-Targeting Cilengitide (L2)

Cilengitide (L2) at either 1 nM or 1 μM led to FAK expression comparable to or slightly higher than in untreated M21 or OV-MZ-6 cells. Also, at both concentrations, within 30 min, p-FAK levels were almost comparable to that in untreated M21 cells but then decreased within 60 min by approximately threefold. In OV-MZ-6 cells, 1 nM of cilengitide (L2) led to an initial approximately 2.2-fold elevation of p-FAK, which then continuously decreased below levels in untreated cells.

#### Integrin αvβ6-Targeting L3

At both concentrations of L3, αvβ6+ HN cells responded with a slight increase in FAK within 60 min, with no effect in OV-MZ-6 cells lacking αvβ6. Levels of p-FAK in HN cells displayed an almost comparable bell-shaped induction at both concentrations, reaching within 30 min up to approximately five- (1 nM) or six-fold (1 μM) elevated levels. A similar but weaker transient FAK activation (approximately 2.9-fold) was noticeable in OV-MZ-6 cells.

#### Integrin α5β1-Targeting L5

L5 at 1 nM increased FAK levels up to approximately 1.6-fold in α5β1+ OV-MZ-6 cells, followed by a continuous decrease below levels in untreated cells. At 1 μM, FAK expression was equal to or only slightly higher than that in untreated cells. The same trend with slightly lower levels was noticed in M21 cells (low α5β1). Also, at both concentrations, FAK activation followed a bell-shaped curve with highest induction at 30 min (1 μM: approximately fourfold; 1 nM: approximately threefold). p-FAK levels in M21 cells were equal or slightly higher than those in untreated cells.

### Impact of Ligands on Integrin-Mediated Cell Migration

**Figure 10:**
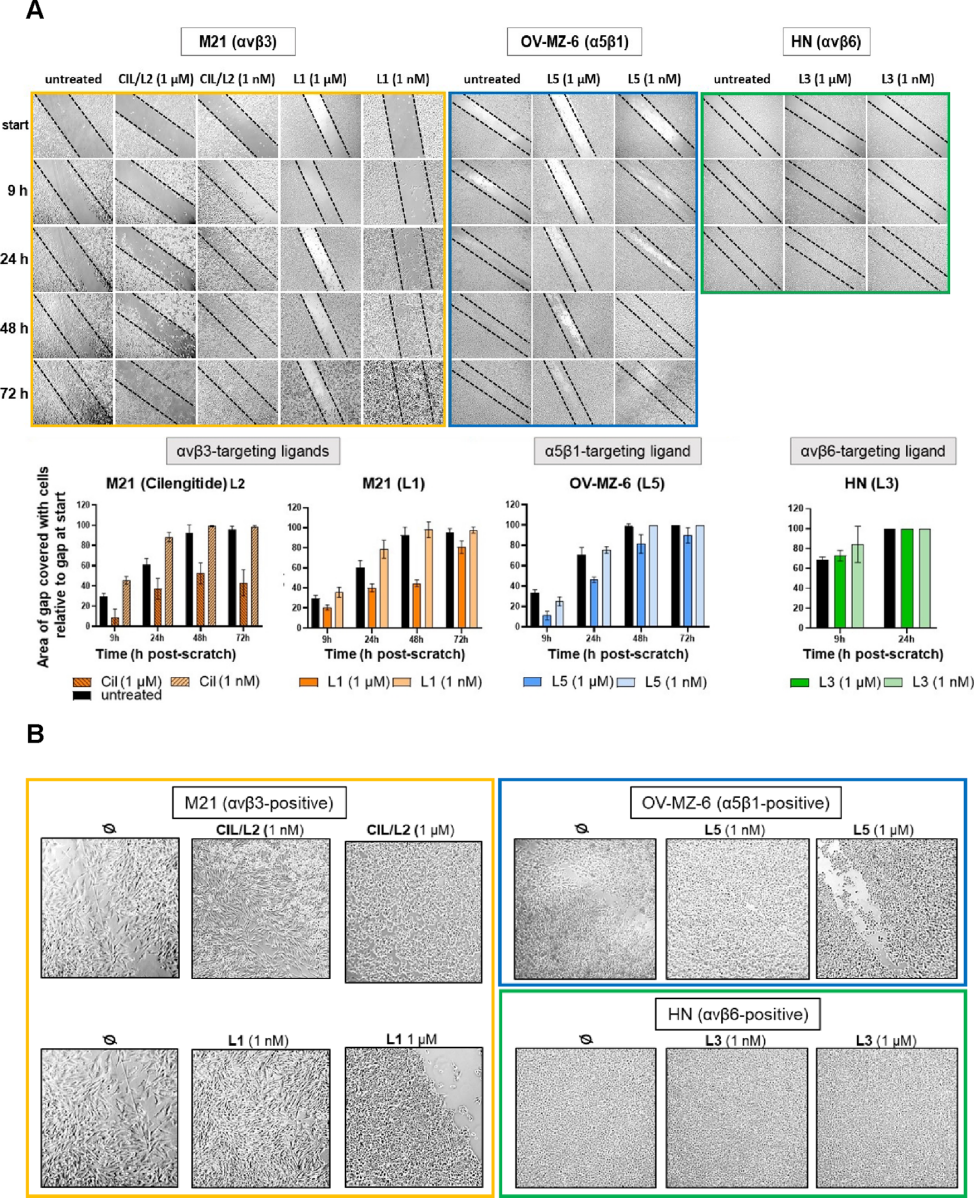
(A) Effect of integrin ligands on cancer cell migration. Integrin αvβ3+ M21 cells, α5β1+ OV-MZ-6, or αvβ6+ HN cells were incubated with 1 μM or 1 nM of cilengitide (L2; αvβ3), L1 (αvβ3), L5 (α5β1), or L3 (αvβ6), and cell migration was monitored by wound scratch assays. Microscopical measurements of wound gaps were taken directly after setting the wound scratch and then after 9, 24, 48, and 72 h, respectively. Wound scratch assays were repeated at least three times. The extent of wound gap closure [%]/cell coverage was calculated by a python script utilizing Canny’s Edge Detection algorithm as described under Materials and summarized in the corresponding histograms (see also Scheme S5 in the Supporting Information). (B) Adhesion of M21, OV-MZ-6, and HN cells in the presence of the respective integrin targeting ligand at low (1 nM) and high (1 μM) concentrations. All cells were incubated for 48 h in the presence of the respective ligand targeting the overexpressed integrin subtype on each cell type (M21/L1 and CIL/L2; OV-MZ-6/L5; (HN/L3)) or left untreated (Ø). Depicted are typical microscopical images of the respective cell monolayers.

As a biological readout for integrin signaling, we analyzed ligand effects on cell adhesion and migration by wound scratch assays (Figure 10A,B). For this, L1 (αvβ3), cilengitide (L2; αvβ3), L3 (αvβ6), or L5 (α5β1) were incubated on αvβ3+ M21 cells, αvβ6+ HN cells, and α5β1+ OV-MZ-6 cells, respectively, at a concentration of 1 μM or 1 nM. Microscopical images were taken directly after wounding the cell monolayers and then after 9, 24, 48, and 72 h, respectively.

Cilengitide (L2) at 1 μM acted antagonistically by abrogating adhesion and spreading of αvβ3+ M21 cells, thereby markedly delaying wound gap closure. Already after 9 h, an approximately threefold reduction of wound gap closure was visible, reaching within 72 h a cell coverage of approximately 40% compared to 95% by untreated cells. The same was observed for OV-MZ-6 cells as control cells, even to a lesser extent (approximately 60% cell coverage within 72 h). At 1 nM, already after 9 h and even stronger after 24 h, cilengitide (L2) provoked an increased wound gap closure by M21 cells, clearly pointing to an agonistic effect, whereas it was not effective in OV-MZ-6 cells when compared to untreated cells (see Scheme S5 in the Supporting Information).

L1 at 1 μM was less effective than cilengitide (L2) in blocking cell migration into the wound gap, still reaching approximately 80% cell coverage within 72 h. Most interestingly, for the migration of OV-MZ-6 cells (low αvβ3), by L1, a more efficient blockade of cell migration than in M21 cells was noticeable, underlining in both cell types an antagonistic ligand behavior. At 1 nM, L1 provoked enhanced wound gap closure of αvβ3+ M21 cells similar to the effect of cilengitide (L2), underscoring its agonistic action, which was, however, not observed for OV-MZ-6 cells with a low αvβ3 expression. L5 at 1 μM blocked migration of α5β1+ OV-MZ-6 cells within 24 h, indicative of an antagonistic ligand action, however, within 72 h, reaching a similar cell coverage like documented for untreated cells. For α5β1– M21 cells, however, no abrogation of cell migration was observed. Treatment of OV-MZ-6 or M21 cells with 1 nM L5 did not alter their migratory capacity. Incubation of αvβ6+ HN cells with L3 displayed neither antagonistic nor agonistic effects on cell migration at either concentration, whereas in αvβ6– OV-MZ-6 cells, a delayed wound gap closure was visible, which, however, joined up with untreated cells after 72 h (see Scheme S5 in the Supporting Information). Since the migratory capability of cells depends on their adhesive activity, in parallel, we checked whether the cells established firm adhesion or were detached as a function of low versus high ligand concentration. Indeed, we found that L1, CIL/L2, and L5 at 1 μM performed as inhibitors of cell attachment correlated with reduced or delayed cell migration. However, at a low ligand concentration, the cells were even more firmly attached and spread, consistent with the observed agonistic ligand behavior and a more efficient wound gap closure than that noticed for untreated cells. In contrast, the adhesive phenotype of HN cells was not changed upon incubation with L3 at both concentrations, going along with no effect of this ligand on cell migration at both concentrations (Figure 10B).

## Discussion and Conclusions

Integrins are valuable drug targets in different pathophysiological events including cancer. The best characterized integrins in cancer are members of the subfamily that recognize the RGD-motif in ECM proteins, including αvβ3, αvβ5, αvβ6, and α5β1. This motivated the development of highly specific and selective ligands as therapeutic and diagnostic tools.^60^ Nonetheless, only a few αvβ3 antagonists have entered clinical trials,^5,7,61,62^ among those cilengitide (L2), which had been tested in multicenter phase II and III clinical trials for antiangiogenic therapy in glioblastoma.^34,37,33,38^ Although its inhibitory efficiency *in vitro* and in preclinical settings had initially raised great hopes for therapy,^63^ it only showed poor clinical efficacy. Soon, it was shown that at a low dose, it rather promoted than inhibited tumor angiogenesis via enhanced VEGF receptor2 recycling and VEGF-stimulated endothelial cell migration. This has been recognized as one of the reasons for cilengitide′s failure in clinical trials for glioblastoma treatment.^28,30−35^

Meanwhile, many studies have unraveled that the local concentration of distinct integrin ligands at diseased cell sites majorly affects ligand performance as either an antagonist/inhibitor at a high dose or as an agonist at a low dose. Upon systemic treatment of patients, possible agonistic ligand behavior is rather unavoidable due to the rapid pharmacokinetics and short half-life of ligands in humans. For cilengitide (L2), it has been shown that it induced structural changes within the heterodimeric integrin molecule, from a compact-closed resting state into an extended-open conformation with dissociated TMDs. This is known to activate the integrin into a highly affine signaling-competent receptor. Within this context, it is interesting to note that FAK, as the most important downstream kinase in the integrin signaling cascade, directly contributes to tumor angiogenesis. Consequently, reduced FAK phosphorylation is linked to constitutively decreased VEGF receptor-2 levels, disturbed integrin activation, and clustering, correlating with a constitutive loss of angiogenic responses.^63−65^ Thus, as agonists, ligands may mimic the action of ECM proteins, thereby triggering integrin-mediated signaling and, in some cases, integrin internalization and tumor biological events arising thereof, like cell adhesion, migration, and proliferation.^66,67,68^ In fact, for a long time, little attention was paid to agonistic ligand performance. At a high dose, ligands may act as antagonists by binding to the integrin, thereby blocking its ECM interaction and thus cell adhesion, signal transduction, and/or integrin internalization.^28,30,32,69−72^

Since this concentration-dependent functional switch of ligand performance has serious consequences for their medical applications, we here aimed to decipher antagonistic from agonistic behavior of a panel of already well-characterized ligands, which selectively target either αvβ3 (L1^39−41^ and cilengitide [L2]), αvβ6 (L3^42^ and L4^43^), or α5β1 (L5^41,44^) in cancer cells (over)expressing the respective integrin subtype. To this end, we examined ligand effects on integrin internalization, as well as cell adhesion and migration, as biological readout for integrin-mediated cell signaling. By monitoring ligand internalization, we proved for all Cy5.5-conjugated ligands considerable integrin subtype-specific cellular uptake. Endocytosis started within 30 min (L1) or was already completed (L3 and L4). To control integrin subtype-selective ligand internalization, we demonstrated minimal or no uptake in tumor cell types that either do not or only weakly express the respective target integrin. This clearly showed the dependence of efficient ligand uptake on a specifically elevated receptor density (see Scheme S2 in the Supporting Information). This type of ligands may represent potential vehicles for integrin-targeted intracellular drug delivery to diseased cell sites, thereby reducing harmful side effects on healthy cells.^73,74^ Besides directly drug-conjugated ligands, several different ligand-coated scaffolds for the transport of encapsulated drugs are currently being explored.^49,75−78^

Since the decisive difference between antagonistic and agonistic ligands lies in the capacity of the latter to trigger integrin activation and signaling at low *substoichiometrical* concentrations,^28,32,70−72,79^ we next studied the expression and activation of the important integrin-related signaling kinases FAK and p44/42^(erk-1/2)^. As a biological measure of ligand-triggered integrin signaling, we then determined the cellular adhesive and migratory phenotype. At a longer ligand incubation time (24 h), cilengitide (L2) was effective in raising the expression and activation of both FAK and p44/42^(erk-1/2)^ in high and low αvβ3-expressing cells. In contrast, at shorter time points at high (1 μM) and low (1 nM) concentrations, cilengitide (L2) displayed only negligible effects on signaling molecules, even decreasing p-FAK over 60 min below levels in untreated cells. Unexpectedly, in low αvβ3-expressing OV-MZ-6 cells, low cilengitide (L2) levels still doubled p-FAK levels, possibly due to its ability to weakly bind to α5β1 (Table 1), which is present in OV-MZ-6 cells in considerable amounts. Thus, the effects of cilengitide (L2) on signaling were not as unambiguous. L1 disclosed similar but weaker effects on signaling kinases but did not elevate p-FAK. It also raised FAK and p-p44/42^(erk-1/2)^ in OV-MZ-6 cells (low αvβ3). Exclusively at a low concentration, L1 provoked a transient and slight increase of FAK in αvβ3 + M21 cells and OV-MZ-6 cells pointing to an agonistic ligand effect. Also, p-FAK expression levels at 1 nM were always prominently higher than those at 1 μM, but not exceeding levels in untreated cells.

Even though only marginal effects of these αvβ3-targeting ligands were noticed on integrin signaling molecules, they corresponded well to the alterations in the cell migratory capacity: cilengitide (L2) acted antagonistically at high concentrations by markedly detaching αvβ3+ M21 cells as well as OV-MZ-6 cells, consequently clearly delaying wound gap closure in the migration assays. In contrast, low cilengitide (L2) accelerated wound gap closure by M21 cells compared to untreated cells, pointing to an αvβ3-dependent agonistic ligand effect. L1 at 1 μM was not quite as effective as cilengitide (L2) in antagonizing M21 cell migration, however, it antagonistically also provoked enhanced wound gap closure by αvβ3+ M21 cells at 1 nM. However, this was not observed for L1 regarding the motility of low αvβ3-expressing OV-MZ-6 cells, indicating a clear αvβ3-specific effect of L1. With respect to the discrepancy between the slight induction of signaling and the marked effects on cell migration, it should be noted that many other factors also influence cell adhesion and migration. As such, cilengitide (L2) was shown to enhance tumor cell invasion by promoting the recycling of αvβ3 and VEGF receptor-2 to the cell membrane, consequently leading to their diminished degradation and thus increased signaling. Concurrently, triggered recruitment of αvβ3 into FAs at the cell periphery was also shown to promote tumor cell migration.^67,68,80^ Thus, for both of these αvβ3-directed integrin ligands, low-dose treatment for inhibitory therapeutic purposes should be avoided since, as already shown for cilengitide (L2), this will lead to the described discouraging clinical results. However, agonistic behavior upon the stimulation of integrin-mediated signaling by those ligands may be re-evaluated and exploited for alternative and novel ligand application scenarios. In case of cilengitide′s low-dose pro-angiogenic and vascular stabilization effects, its use in cancer therapy was soon repurposed, by showing that it strengthened immature and leaky tumor vessels, thereby improving tumor perfusion and thus drug delivery, as well as immune cell recruitment and reduced tumor cell extravasation.^36,81^

In contrast, after long incubation times, αvβ6-targeting ligand L3 was not effective in modulating signaling molecules in either cell type. However, within 60 min, at high and low concentrations, αvβ6+ HN cells responded with a slight FAK increase, whereas no effect was noticeable in OV-MZ-6 cells, which lack αvβ6. However, a pronounced bell-shaped induction of p-FAK was observed in HN cells at both ligand concentrations, with a similar but weaker pattern also detected in OV-MZ-6 cells. Despite putative integrin signaling activation, L3 at both ligand concentrations displayed neither an antagonistic nor an agonistic impact on wound gap closure by αvβ6+ HN cells. This was quite surprising since it is known that αvβ6 orchestrates and activates signaling events in favor of increased cell motility via associating with the Fyn kinase, followed by FAK recruitment and activation of the Raf-Erk-MAPK pathway.^5^ However, one has to take into account that in contrast to other members of the RGD-recognizing integrin subfamily, αvβ6 is operative in alternative signaling mechanisms via affecting TGF-β1 activation upon its binding to the RGD-motif contained within the LAP linked to inactive TGF-β1.^82^ As long as LAP is noncovalently linked to inactive TGF-β1, the binding of the latter to its receptors is abrogated and signaling prevented. Indeed, for oral squamous cell carcinoma (OSCC) cells, it was shown that αvβ6 binding to LAP enhanced cell migration.^5^ Thus, the findings for L1 and cilengitide (L2) differ from that found for L3 and also the data from migration assays did not indicate antagonistic or agonistic performance of L3. This suggests that such a ligand may be, e.g., suitable for nuclear imaging of αvβ6-expressing cancer cells,^78^ most prominently head and neck squamous cell carcinoma (HNSCC) or pancreatic cancer cells overexpressing this integrin subtype. Indeed, several αvβ6-targeting radiolabeled tracers have already shown promise in preclinical positron emission tomography (PET) and single photon emission computed tomography (SPECT) imaging studies and their applicability for cancer diagnosis and radiotherapy.^83−86^

The integrin α5β1-targeting ligand L5 did not modulate (p-)FAK and (p-)p-44/42^(erk-1/2)^ in OV-MZ-6 cells after long-time ligand incubation, while unexpectedly increasing (p-)p-44/42^(erk-1/2)^ in M21 cells, which harbor small amounts of this integrin (see Schemes S3 and S4 in the Supporting Information) (Figure 8). At shorter incubation times, L5 at 1 nM increased FAK levels in α5β1+ OV-MZ-6 cells, followed by a continuous decrease below the levels in untreated cells. At 1 μM L5, FAK expression was equal to or only slightly higher than that in untreated cells. A similar trend with slightly lower levels was observed in the M21 cells. Additionally, at both concentrations, FAK activation followed a bell-shaped curve with considerable induction, while in M21 cells, it was either equal to or slightly higher. These transient changes did not match with the noticed alterations in the cell migratory capacity: exclusively at a high L5 concentration, diminished migration of α5β1+ OV-MZ-6 cells was noticeable, indicative of antagonistic ligand behavior, whereas it did not alter the cell migratory capacity at 1 nM, despite its positive effect on FAK activation. This indicated the missing agonistic behavior of this ligand.

In conclusion, the findings of this study underscore the need for an in-depth evaluation of integrin ligand performance prior to their medical use. This is thought to allow for the identification of the most promising ligands for their most suitable clinical application. This will enable the exploitation of their full potential in a plethora of new arising opportunities, as either therapeutic compounds, target-specific drug-delivery vehicles, or diagnostic tools.^7,28,32,36^ Meanwhile, substantial efforts have been made to better understand and unravel the molecular determinants and conformational rearrangements during the mechanism of integrin activation. Also, the complex pharmacology of the ligands, their *in vivo* half-life, integrin subtype specificity, selectivity, and dilution in the bloodstream upon systemic application becomes more and more clear. Moreover, advances in the rational design of new integrin ligands were inspired to develop either pure inhibitors or agonists. Indeed, as alternatives to ligands targeting the ECM binding site within integrins, the design of allosteric ligands is currently being considered for antiangiogenic cancer therapies, aiming at stabilizing the integrin in its inactive conformation, e.g., by targeting the cytoplasmic integrin tail.^87^ Indeed, more recently, several publications have reported on the development of those “pure” integrin antagonists that do not activate the targeted integrins. This will finally achieve therapeutic value for an extended repertoire of medical integrin ligand use.^28,88−91^

## Experimental Section

### Materials

#### Chemicals

Unless otherwise noted, all reagents, solvents, and resins were obtained from commercial suppliers, of analytical, “for synthesis”, peptide, or HPLC grade, and used without further purification. L4 was assembled using a Macherey-Nagel CHROMABOND vacuum manifold and an ultrasonic bath SONOREX RK 52 H (interior dimensions 150 × 140 × 100 mm and operating volume 1.2 L) by BANDELIN electronic (Germany). Saturated solutions were aqueous solutions.

#### Cell Culture Materials

##### *In Vitro* Cell Experiments

Trypsin-EDTA (10×), Dulbecco’s Modified Essential Medium GlutaMAX (DMEM), Roswell Park Memorial Institute-1640 Medium (RPMI-1640), Minimum Essential Medium (MEM), fetal calf serum (FCS), and phosphate buffer saline (PBS; pH 7.4) were purchased from Gibco, Thermo Fisher Scientific (Carlsbad, California, USA). CellMask Green Plasma Membrane Stain (no. C37608) and Hoechst 33342 (no. H3570) were obtained from Thermo Fisher Scientific.

#### Antibodies

For integrin binding affinity assay, the following reagents were used: integrin αvβ3: (1) 1.0 μg/mL human vitronectin (Merck Millipore, Darmstadt, Germany; #CC080), (2) 2.0 μg/mL human αvβ3 (R&D, Minneapolis, Minesotta, USA; #3050-AV-050). (3) 2.0 μg/mL mouse-anti-human CD51/61 (BD Biosciences, Franklin Lakes, New Jersey, USA; #555504). (4) 2.0 μg/mL anti-mouse IgG-POD (Sigma-Aldrich, #A9044-2ML). Integrin αvβ6: (1) 0.4 μg/mL LAP (TGF-β) (R&D, #246-LP-025), (2) 0.5 μg/mL human αvβ6 (R&D, #3817-AV-050), (3) 1:500 dilution mouse-anti-human αv (MAB1978) (Merck Millipore, #MAB1978), (4) 2.0 μg/mL anti-mouse IgG-POD (Sigma-Aldrich, #A9044-2ML). Integrin α5β1: (1) 0.5 μg/mL human fibronectin (Sigma-Aldrich, #F0895-1MG), (2) 2.0 μg/mL human α5β1 (R&D, 3230-A5-050), (3) 1.0 μg/mL mouse-anti-human CD49e (BD Biosciences, #555651), (4) 2.0 μg/mL anti-mouse IgG-POD (Sigma-Aldrich, #A9044-2 ML). For Western blot analysis, the following antibodies were used: (p-)p44/42^(erk-1/2)^ (1) 1:300 dilution of rabbit-anti-human p44/42^(erk-1/2)^ antibody (Cell Signaling Technology, Frankfurt, Germany, #9102) in 3% (w/v) bovine serum albumin (BSA)/TBS-T, (2) 1:5,000 dilution goat-anti-rabbit horseradish-conjugated secondary antibody in 1% (w/v) BSA/TBS-T. Phospho-p44/42^(erk-1/2)^: (1) 1:200 dilution rabbit-anti-human p44/42^(erk-1/2)^ (Thr202/Tyr204) antibody (Cell Signaling Technology, #9101) in 3% (w/v) BSA/TBS-T, (2) 1:5,000 dilution goat-anti-rabbit horseradish-conjugated secondary antibody in 1% (w/v) BSA/TBS-T. FAK: (1) 1:300 dilution mouse-anti-human FAK antibody (Cell Signaling Technology, #9102) in 3% (w/v) BSA/TBS-T, (2) 1:5,000 dilution goat-anti-mouse horseradish-conjugated secondary antibody in 1% (w/v) BSA/TBS-T. Phospho-FAK: (1) 1:300 dilution mouse-anti-human p-FAK antibody (Cell Signaling Technology) in 3% (w/v) BSA/TBS-T, (2) 1:5,000 dilution goat-antimouse horseradish-conjugated secondary antibody in 1% (w/v) BSA/TBS-T. Differences in blotting efficiency and protein loading were normalized by using a primary antibody against glyceraldehyde-3-phosphate dehydrogenase (GAPDH, 1:10,000 dilution in 1% (w/v) BSA/TBS-T, at RT for 45 min (Millipore, Schwalbach, Germany) and a secondary horseradish-conjugated goat-anti-mouse antibody (1:10,000 dilution in 1% (w/v) BSA/TBS-T, 40 min at RT).

### Peptide Synthesis

#### Chromatography

Analytical high-pressure chromatography electrospray ionization-mass spectrometry (HPLC-ESI-MS) was performed on an UltiMate 3000 UHPLC (Dionex) equipped with a LCQ Fleet mass spectrometer (Thermo Scientific) using a long C18 column Hypersil Gold aQ 175 Å, 3 μm, 150 mm × 2.1 mm) or a short C18 column (“S2″, Accucore C18, 80 Å, 2.6 μm, 50 × 2.1 mm) from Thermo Scientific. Linear gradients (0.9 mL/min; 8 min, S1 or 5 min, S2) of H_2_O (0.1% v/v formic acid) and acetonitrile (MeCN; 0.1% v/v formic acid) were used for analytical purposes. Analytical HPLC-MS spectrometry of cilengitide (L2) was performed on a Vanquish Horizon Flex UHPLC system (Thermo Scientific) using a long C18 column (Hypersil Gold aQ 175 Å, 3 μm, 150 × 2.1 mm) from Thermo Scientific. A linear gradient (0.9 mL/min, 15 min) of H_2_O (0.1% v/v formic acid) and acetonitrile (MeCN; 0.1% v/v formic acid) was used (10–90% MeCN over 15 min). Analytical analysis via HPLC confirmed a purity of ≥95% (220, 250, 272, and/or 214 nm). Semipreparative reversed-phase HPLC was performed using a Waters instrument including a Waters 2545 (Binary Gradient Module), Waters SFO (System Fluidics Organizer), Waters 2996 (Photodiode Array Detector), and Waters 2767 (Sample Manager). Suitable linear gradients of H_2_O (0.1% v/v trifluoroacetic acid (TFA), buffer A) and MeCN (0.1% v/v TFA, buffer B) were applied for the purification of all compounds using (unless otherwise noted) a C18 column (Reprosil 100 C18, 5 μm, 150 × 30 mm, Dr. Maisch) with a flow rate of 40 mL/min. Column chromatographic separations were carried out with a 100-fold mass excess of silica gel (40–63 μm, Si 60, 230–400 mesh ATSM, Merck Millipore) at 1 bar overpressure. L4 was purified by preparative HPLC (Shimadzu HPLC system) equipped with a C18-bounded preparative RP-HPLC column (Phenomenex Kinetex 21.2 × 150 mm, 5 μm).

#### NMR

^1^H NMR spectra were recorded at 298 K on a 400 MHz AV spectrometer from Bruker. Chemical shifts are given in *parts per million* (ppm). Internal standards for the chemical shifts of ^1^H were the following solvent signals: DMSO-*d*_6_: 2.50 ppm (^1^H NMR) and 39.52 ppm (^13^C NMR). Abbreviations for NMR signal multiplicities are singlet (s), doublet (d), triplet (t), quartet (q), and multiplet (m). Coupling constants *J* are given in Hz as mean values of found experimentally values. The data were processed and evaluated using MestreNova (version 9.0).

#### General Procedures

##### GP1 Loading of TCP-Resin

Peptide synthesis was carried out using 2-chlorotrityl chloride polystyrene resin (2-CTC resin; 1.03 mmol/g; 1.00 equiv, Carbolution Chemicals, St. Ingbert, Germany) using standard Fmoc peptide synthesis conditions. The resin was first treated with Fmoc-Xaa-OH (1.20 equiv) and *N*,*N*-diisopropylethylamine (DIPEA; 2.50 equiv) in anhydrous dichloromethane (DCM; 10 mL/g of resin) at room temperature (RT) for 2 h. Unconjugated 2-chlorotrityl chloride groups were capped by the addition of a solution of MeOH and DIPEA (5:1; v/v; 1.0 mL/g of resin) for 15 min. After filtration, the resin was washed with CH_2_Cl_2_ (4×) and *N,N*-dimethylformamide (DMF; 5×). The loading capacity was estimated to be 1.03 mmol/g (100%).

##### GP2 on-Resin Fmoc Deprotection

The N-terminal, Fmoc-protected resin-bound peptide was treated for 10 min and then 15 min with a solution of piperidine in DMF (20%; v/v). Afterward, the resin was washed with DMF (8×).

##### GP3 Standard on-Resin Amino Acid Coupling

Fmoc-Xaa-OH (2.00 equiv), *O*-(7-azabenzotriazol-1-yl)-*N*,*N*,*N*′,*N*′-tetramethyluronium-hexafluorphosphate (HATU) (2.0 equiv), 1-hydroxybenzotriazole (HOBt; 2.00 equiv), and DIPEA (5.00 equiv) in DMF (7 mL/g resin) were added to the resin-bound Fmoc-deprotected, free amine peptide and shaken for 1 h at RT. Afterward, the resin was washed with DMF (5×).

##### GP4 on-Resin *N*-Methylation^92,93^

The linear, Fmoc-deprotected peptide was washed with DCM (3×) and subsequently incubated with a solution of 2-nitrobenzenesulfonyl chloride (o-Ns-Cl, 4.00 equiv) and 2,4,6-collidine (10.0 equiv) in DCM for 20 min at RT. After washing the resin with DCM (3×) and THF abs. (5×), a solution containing PPh_3_ (5.00 equiv) and MeOH abs. (10.0 equiv) in THF abs. was added. DIAD (5.00 equiv) in a small amount of THF abs. was added stepwise to the resin. The solution was incubated for 15 min and washed with THF (5×) and DMF (5×). For o-Ns deprotection, the resin-bound o-Ns peptides were stirred in a solution of mercaptoethanol (10.0 equiv) and DBU (5.00 equiv) in DMF (10 mL/g resin) for 5 min. The deprotection procedure was repeated one more time. Finally, the resin was washed with DMF (5×).

##### GP5 Cleavage of Linear Peptides from the Resin

First, the resin was washed with DCM (3×). Subsequently, for a complete cleavage from the resin, the peptides were treated with a solution of DCM and hexafluoroisopropanol (HFIP; 4:1; v/v; 3×) for 30 min. Finally, the solvent was evaporated under a reduced pressure.

##### GP6 Peptide Cyclization

After dissolving the linear peptide in N,N dimethyl formamid (DMF) (3.30 mM), NaHCO_3_ (5.00 equiv) and diphenylphosphoryl azide (3.00 equiv) were added. The resulting solution was stirred at RT overnight or until no linear peptide could be observed by HPLC-ESI-MS.

##### GP7 Saponification of Methyl Esters

The starting material was first dissolved in MeOH/H_2_O (3:1), and subsequently LiOH (5.00 equiv, 50 mM) was added. The reaction mixture was stirred overnight (appr. 18 h) at RT. Afterwards, either extraction with EtOAc (3×) and 1 N HCl (1×) or, in the case of end products, purification via semipreparative RP-HPLC was performed.

##### GP8 Standard in-Solution Amino Acid Coupling

The acid (1.20 equiv), HATU (1.20 equiv), HOBt (1.20 equiv), and DIPEA (5.00 equiv) in DMF (∼0.10 mol/L) were preincubated for 15 min at RT. After addition of the free amine (1.0 equiv), the solution was stirred overnight at RT. After removal of the solvent under reduced pressure, the residue was redissolved in EtOAc and washed with sat. NH_4_Cl (1×), sat. NaHCO_3_ (1×), and brine (1×). Finally, after the organic phase was dried with Na_2_SO_4_, the solvent was removed *in vacuo*, yielding a crude peptide conjugate. The isolated product was used directly in the next reaction step.

##### GP9 Boc, Trityl, Pbf, *^t^*Bu Deprotection in Solution

After dissolving the starting material in a solution of TFA/TIPS/H_2_O/DCM (85:2.5:2.5:10; v/v/v/v), the resulting solution was stirred for 30 min (reaction progress monitored by HPLC-ESI-MS) at RT. Subsequently, the reaction was quenched with toluene, and the solvent was evaporated under reduced pressure. The hereby obtained oily residue was precipitated in diethyl ether (Et_2_O).

#### Synthesis of (*S*)-4-(4-(3-((4-Methoxypyridin-2-yl)amino)propoxy)phenyl)-3-(4-(3-(pent-4-ynamido)propoxy)benzamido)butanoic Acid (L1)

Ligand L1 ((*S*)-4-(4-(3-((4-methoxypyridin-2-yl)amino)propoxy)phenyl)-3-(4-(3-(pent-4-ynamido)propoxy)benzamido)-butanoic acid) was prepared according to the literature.^50^ In particular, the following reaction steps were performed.^40,41,44,49,50^ Starting from (S)-methyl-3-(*tert*-butoxycarbonylamino)-4-(4-(3-(4-methoxypyridin-2-ylamino)propoxy)phenyl)butanoate^41^ (20 mg, 42.3 μmol, 1.00 equiv), the following reaction sequence was performed: after Boc deprotection (GP9), 4-(3-((*tert*-butoxycarbonyl)amino) propoxy)benzoic acid (15 mg, 50.7 μmol, 1.20 equiv) was coupled according to GP8. The resulting crude product SI-1 was again Boc-deprotected (GP9) and coupled to 4-pentynoic acid (GP 8; 4.45 mg, 45.3 μmol, 1.20 equiv). Saponification according to GP7 and semipreparative RP-HPLC purification (20–60%, buffer B, 15 min) resulted in L1 (5.87 mg, 9.51 μmol, 21%) as yellowish solid. RP-HPLC (5–95%, 5 min): *t*_R_ = 2.40 min. MS (ESI, positive): *m*/*z* = 617.30 [M+H^+^].

#### Synthesis of Cilengitide/c(RGDf(*N*Me)V) (L2)

Cilengitide (L2) was prepared according to the literature.^54,93^

#### Synthesis of c(FRGDLAFp(*N*Me)K) (L3)

The ανβ6-targeting ligand L3 was synthesized according to a literature procedure.^42,49^ RP-HPLC (5–95%, 5 min): *t*_R_ = 2.78 min. **MS** (ESI, positive): *m*/*z* = 1046.52 [M+H+], 524.1 [M/2+H+]. ^**1**^**H NMR** (400 MHz, DMSO-*d*6): δ [ppm] = 8.69–8.54 (m, 2H), 8.03 (d, *J* = 7.2 Hz, 1H), 7.92–7.83 (m, 1H), 7.69–7.58 (m, 4H), 7.52–7.38 (m, 3H), 7.34 (d, *J* = 7.5 Hz, 1H), 7.32–7.17 (m, 11H), 7.08 (d, *J* = 8.1 Hz, 2H), 6.98 (t, *J* = 7.3 Hz, 1H), 5.60–5.40 (m, 1H), 4.89–4.75 (m, 3H), 4.58–4.46 (m, 2H), 4.42–4.31 (m, 2H), 3.96 (dd, *J* = 15.5, 4.8 Hz, 1H), 3.73 (dd, *J* = 15.6, 5.5 Hz, 1H), 3.15–3.04 (m, 2H), 3.04–2.90 (m, 3H), 2.82 (d, *J* = 12.5 Hz, 1H), 2.76 (s, 1H), 2.72–2.63 (m, 3H), 2.42 (s, 2H), 2.07 (s, 3H), 2.05–1.94 (m, 2H), 1.85–1.76 (m, 1H), 1.73–1.61 (m, 3H), 1.57–1.37 (m, 7H), 1.30–1.16 (m, 4H), 1.13–1.00 (m, 2H), 0.92–0.79 (m, 6H).

#### Synthesis of c(RGD-Chg-isoE)-CONH_2_ (L4)

The ανβ6-targeting ligand L4 was synthesized according to a previously published protocol.^43,49^ RP-HPLC (0–90%, 20 min, S42): *t*_R_ = 13.65 min. MS (ESI, positive): *m*/*z* = 596.52 [M+H+].

#### Synthesis of (*S*)-2-(2,6-Dimethyl-4-(3-(pent-4-ynamido)propoxy)benzamido)-3-(2-(3-guanidinobenzamido)acetamido)propanoic Acid (L5)

Ligand L5 was synthesized according to a literature procedure.^58^ In particular, following the Boc strategy, it was synthesized in-solution and prepared from *^t^*Bu-deprotected (GP9) benzyl 4-(4-(*tert*-butoxycarbonyl)-3,5-dimethylphenoxy)butylcarbamate^41^ (20 mg, 56 μmol, 1.05 equiv) in compliance with the following reaction sequence: coupling of H-l-Dap(Boc)-OMe (GP8), Boc deprotection (GP9), coupling of Boc-Gly-OH (GP8), Boc deprotection (GP9), coupling of (*Z*)-3-(2,3-Bis(*tert*-butoxycarbonyl)guanidino)benzoic acid^49^ (GP8), Cbz deprotection with H_2_/Pd/C in MeOH for 3 h, coupling of 4-pentynoic acid (GP9), and global deprotection according GP7 and GP9. Purification via semipreparative RP-HPLC (20–60% buffer B, 15 min) led to compound L5 (10.3 mg, 17 μmol, 32%) as white solid. RP-HPLC (5–95%, 8 min): *t*_R_ = 3.23 min. MS (ESI, positive): *m*/*z* = 608.1 [M+H^+^].

#### Synthesis of the Cy5.5-Conjugates of L1, L3–L5

L1-Cy5.5, L3-Cy5.5, L4-Cy5.5, and L5-Cy5.5 were synthesized according to the same procedure as previously published.^49^ In particular, L1-Cy5.5 to L5-Cy5.5 were synthesized according to the following protocol: in a first step, Boc-6-Ahx–OH (1.20 equiv) was coupled to L1, L3, L4, or L5 (GP8), yielding the Ahx-extended version of the ligands. After complete deprotection and purification via RP-HPLC, Cy5.5-NHS ester (2 mg, 2.61 μmol, 1.00 equiv) was conjugated to the Ahx extended version (1.00 equiv) in DMF. After addition of DIPEA (3.00 equiv), the solution was stirred for 3 h at RT in the dark. The progress of the reaction was monitored by HPLC-ESI-MS. After the completion of the reaction, the solvent was removed under reduced pressure. The crude product was purified via semipreparative reversed phase HPLC (55–95% buffer B, 15 min).

L1-Cy5.5: RP-HPLC (5–95%, 5 min): *t*_R_ = 4.35 min. MS (ESI, positive): *m*/*z* = 1214.4 [M], 608.2 [M/2+H^+^].

L3-Cy5.5: RP-HPLC (5–95%, 5 min): *t*_R_ = 4.56 min. MS (ESI, positive): *m*/*z* = 1724.5 [M+H^+^], 863.0 [M/2+H^+^].

L4-Cy5.5: RP-HPLC (5–95%, 5 min): *t*_R_ = 4.40 min. MS (ESI, positive): *m*/*z* = 1401.5 [M], 701.9 [M/2+H^+^].

L5-Cy5.5: RP-HPLC (5–95%, 5 min): *t*_R_ = 4.56 min. MS (ESI, positive): *m*/*z* = 1205.4 [M], 603.6 [M/2+ H^+^].

#### Determination of Integrin Ligand Binding Specificity and Subtype-Selective Affinity by an ELISA-Type Solid-Phase Binding Assay

The integrin binding assay was done to assess the specificity and subtype-selective affinity of L1–L5 and their respective Cy5.5-conjugated ligands by using isolated and purified integrins in cell-free ligand binding assays, as previously published.^59^Table 2 summarizes the used ECM ligands, integrins, and antibodies. In brief, flat-bottom 96-well plates were coated overnight at 4 °C with the natural ECM integrin ligands, vitronectin (for αvβ3-directed ligands), fibronectin (for α5β1-directed ligands), or LAP of TGF-β1 (for αvβ6-directed ligands) (100 μL/well in buffer containing 15 mM Na_2_CO_3_, 35 mM NaHCO_3_, pH 9.6). Subsequently, each well was washed in PBS, 0.01% (v/v) Tween 20, 137 mM NaCl, 2.7 mM KCl, 10 mM Na_2_HPO_4_, 2 mM KH_2_PO_4_, pH 7.4 (PBS-T), and blocked for unspecific binding events for 1 h at RT in 20 mM Tris–HCl, pH 7.5, 150 mM NaCl, 1 mM CaCl_2_, 1 mM MgCl_2_, 1 mM MnCl_2_, and 1% (w/v) BSA. Meanwhile, dilution series of the different integrin ligands and the internal standards were prepared, ranging from 20 μM to 256 pM in 1:5 dilution steps. After washing the assay plate three times in PBS-T, 50 μL of the ligand dilution series was transferred to each well in six appropriate concentrations. Then, 50 μL of a solution of the respective human integrin (2) was transferred to the wells and incubated for 1 h at RT. The better the ligands bind the integrin, the more they inhibit binding of the integrin to the underlying immobilized ECM protein. The plate was washed in PBS-T buffer, followed by the addition of the primary antibody (3). After incubation for 1 h at RT, the plate was washed in PBS-T prior to the addition of a secondary peroxidase-conjugated antibody (4) for 1 h at RT. After repeated washes in PBS-T, the plate was developed by the addition of the chromogenic substrate 3,3‘,5,5‘-tetramethylbenzidine (TMB) (SeramunBlue, Seramun Diagnostic GmbH) and incubated in the dark for approximately 1–5 min at RT. In the presence of a peroxidase, oxidation of TMB leads to a blue-colored solution. The reaction was stopped in 3 M H_2_SO_4_ and the absorbance spectrophotometrically detected at 450 nm. The IC_50_ value (with 95% confidence interval) of each integrin ligand resulted from a sigmoidal fit of two data rows (serial dilution rows) performed by the OriginPro 9.0G statistical software. All IC_50_ values were referenced to the affinity of the internal standards: cilengitide (L2) (IC_50_ value for αvβ3: 0.54 nM and for α5β1:15.4 nM) and the linear peptide RTDLDSLRT for αvβ6 (IC_50_ value: 29.5 nM). The IC_50_ values for all ligands and their respective Cy5.5-labeled counterparts are given in Table 1.

**Table 2:** Antibodies, Integrins, and ECM Proteins Used in the ELISA-Type Ligand Binding Assay

**integrin**	**antibody/integrin/ECM protein**	**supplier/catalog number**	**concentration**[μg/mL]
**αvβ3**	**(1)** human vitronectin	Merck Millipore, #CC080	1.0
	**(2)** human αvβ3-integrin	R&D, #3050-AV-050	2.0
	**(3)** mouse-anti-human CD51/61	BD Biosciences, #555504	2.0
	**(4)** anti-mouse IgG-POD	Sigma-Aldrich, #A9044	2.0
**α5β1**	**(1)** human fibronectin	Sigma-Aldrich, #F0895–1MG	0.5
	**(2)** human α5β1-integrin	R&D, 3230-A5–050	2.0
	**(3)** mouse-anti-human CD49e	BD Biosciences, #555651	1.0
	**(4)** anti-mouse IgG-POD	Sigma-Aldrich, #A9044	2.0
**αvβ6**	**(1)** LAP (TGF-β1)	R&D, #246-LP-025	0.4
	**(2)** human αvβ6-integrin	R&D, #3817-AV-050	0.5
	**(3)** mouse-anti-human αv	Merck Millipore, #MAB1978	1:500 dilution
	**(4)** anti-mouse IgG-POD	Sigma-Aldrich, #A9044	2.0

#### Cell Culture

Cell cultivation and handling were performed as previously published.^49^

#### Time-Dependent Cellular Uptake of Cy5.5-Labeled Integrin Ligands

Microchamber cell culture slides were coated with fibronectin (2 μg/mL), followed by cell seeding at a density of 50,000 cells/well. After 24 h of cultivation, cells were treated with integrin ligands L1-Cy5.5 to L5-Cy5.5 (1 μM in 0.01% (w/v) CaCl_2_ and 0.1% (w/v) BSA in PBS; 250 μL/well) for 3, 30, 60, or 300 min at 37 °C. The cells were washed in PBS/0.01% (w/v) CaCl_2_ (6 × 800 μL) and the fluorescence signal intensity recorded using the LSM 700 microscope (Zeiss, Jena, Germany). CellMask Green Plasma Membrane Stain was used to stain the cell membrane; nuclei were stained with Hoechst 33342.

#### Western Blot Analysis

The kinases (p-)p44/42^(erk-1/2)^ and (p-)FAK were detected as previously published.^15,8^ Briefly, cells were grown for 18 h on six-well plates in DMEM (0.45 Mio cells/well). After medium exchange, fresh medium with L1–L5 (concentration: 1.0 μM, 2 mL/well) was added, and cells were incubated for another 24 h at 37 °C. In a second series, cells were incubated with L1–L5 (1 μM or 1 nM) for 5, 30, or 60 min. Thereafter, the cellular proteins were extracted and processed by Western blotting. Proteins were visualized using the Pierce ECL chemiluminescent substrate (Thermo Fisher Scientific). Band signal intensities for (p-)p44/42^(erk-1/2)^ and (p-)FAK were evaluated using the Bio-Rad Imager Gel Doc XR + and ChemiDoc XRS + Systems and the software Image Lab. Differences in protein loading and blotting efficiency were normalized by reprobing the membranes with a monoclonal antibody directed to GAPDH. Signal intensities for protein bands of interest were normalized to those recorded for GAPDH. Typical Western blot images are depicted together with histograms summarizing the mean values of normalized fluorescence signal intensities as *n*-fold, by setting those obtained for untreated cells to “1”.

#### Wound Scratch Cell Migration Assay

The assays were performed according to a modified protocol as previously published.^94^ Cells were cultivated on fibronectin-coated 12-well cell culture plates until cell monolayers reached an approximately 80% confluency. Then, the wound scratch was set utilizing a 200 μL pipet tip. Subsequently, fresh culture medium was added in the absence or presence of L1–L5 (concentration: 1 μM or 1 nM), and microscopical images (Zeiss, Oberkochen, Germany) were taken at two identical positions of the wound gap directly after wounding and then at 9, 24, 48, or 72 h of further cell cultivation. Each wound scratch assay was repeated three times. A custom python script was implemented to analyze wound gap closure over time: after cropping the images, we used the OpenCV^95^ implementation of Canny’s Edge Detection Algorithm^96^ to ensure reliable measurements even with variable lighting conditions.^97^ As a first step, a 5 × 5 Gaussian filter was applied to remove background noise. The smoothened images were then filtered by a Sobel kernel^98^ and the magnitude and direction of the intensity gradient of the images calculated. Then, a non-maximum suppression algorithm was applied to identify relevant pixels that presumably contribute to edges. In a final step, threshold hysteresis was utilized to distinguish true edges from variations in intensity or the remaining background noise. The resulting images were then segmented and binarized by use of a grid filter and analyzed with respect to cell-covered area relative to the start image of each series.

## Supporting Information Available

(Scheme S1) HPLC-MS/NMR spectra of L1–L5; (Scheme S2) controls to integrin-specific cellular uptake of Cy5.5-labeled integrin ligands; (Scheme S3) identification of cancer cell types overexpressing the respective targeted integrin subtype; (Scheme S4) controls to the immunostaining depicted in Scheme S1 in the absence of the primary integrin-directed antibodies to determine possible unspecific staining of the Alexa-568-labeled secondary antibody; (Scheme S5) effect of integrin ligands on cancer cell migration (control cells) to Figure 10A in the main manuscript) (PDF)
List of compounds (CSV)

## Abbreviations

CLSM: confocal laser scanning microscopy
ECM: extracellular matrix
Erk: extracellular regulated kinase
FA: focal adhesion
FAK: focal adhesion kinase
GAPDH: glyceraldehyde-3-phosphate dehydrogenase
LAP: latency-associated peptide (of TGF-β)
MAPK: mitogen-activated protein kinases
p-FAK: phospho-FAK
Raf: rapidly accelerated fibrosarcoma
TMD: transmembrane domain
VEGF: vascular endothelial growth factor
