# Supplementary material for: Switching Roles—Exploring Concentration-Dependent Agonistic versus Antagonistic Behavior of Integrin Ligands
Source: J Med Chem. 2025 Feb 5;68(4):4334–51. doi: 10.1021/acs.jmedchem.4c02111 (PMC11874007; doi:10.1021/acs.jmedchem.4c02111)
Supplement: Supplementary file 1 — jm4c02111_si_001.pdf [file jm4c02111_si_001.pdf]

SUPPORTING INFORMATION

Switching roles – exploring concentration-dependent agonistic versus antagonistic behavior of integrin ligands

Beatrice Stefanie Ludwig[1,2], Nils Krautkremer[3], Stefano Tomassi[4], Salvatore Di Maro[5], Francesco Saverio Di Leva[4], Anke Benge[6], Markus Nieberler[3], Horst Kessler[7], Luciana Marinelli[4], Susanne Kossatz[1,2,8], Ute Reuning[6]*

[1] TUM University Hospital, Technical University of Munich, Klinikum rechts der Isar, School of Medicine & Health, Department of Nuclear Medicine, Ismaninger Strasse 22, 81675 Munich, Germany.
[2] TUM University Hospital, Technical University of Munich, Klinikum rechts der Isar, School of Medicine & Health, Central Institute for Translational Cancer Research (TranslaTUM), Ismaninger Strasse 22, 81675 Munich, Germany.
[3] TUM University Hospital, Technical University of Munich, Klinikum rechts der Isar, School of Medicine & Health, Department of Oral and Maxillofacial Surgery, Ismaninger Strasse 22, 81675, Munich, Germany.
[4] University of Naples Federico II, UNINA – Department of Pharmacy, Via Domenico Montesano 49, 80131 Naples, Italy.
[5] Università degli Studi della Campania “Luigi Vanvitelli”, SUN – Department of Environmental, Biological and Pharmaceutical Sciences and Technologies, Viale Abramo Lincoln, 5, 81100 Caserta, Italy.
[6] TUM University Hospital, Technical University of Munich, Klinikum rechts der Isar, School of Medicine & Health, Department of Obstetrics & Gynecology, Clinical Research Unit, Ismaninger Strasse 22, 81675 Munich, Germany.
[7] Institute for Advanced Study, Department of Chemistry, School of Natural Sciences and Bavarian NMR Center (BNMRZ), Technical University Munich, Lichtenbergstraße 2a, 85748 Garching, Germany.
[8] Department of Chemistry, School of Natural Sciences, Technical University Munich, Ismaninger Strasse 22, 81675 Munich, Germany.

*Corresponding author

TABLE OF CONTENTS

Supplementary schemes (pp. S2-S10) (PDF)

Scheme S1. HPLC-MS/NMR spectra of L1-L5 S2-S7

Scheme S2. Controls to integrin-specific cellular uptake of Cy5.5-labeled integrin ligands S8

Scheme S3. Identification of cancer cell types overexpressing the respective targeted integrin subtype (PDF) S9

Scheme S4. Controls to the immunostaining depicted in Scheme S3 in the absence of the primary integrin-directed antibodies to determine possible unspecific staining of the Alexa-568-labeled secondary antibody (PDF) S9

Scheme S5. Effect of integrin ligands on cancer cell migration (control cells) to Fig. 10A in the main manuscript) (PDF) S10

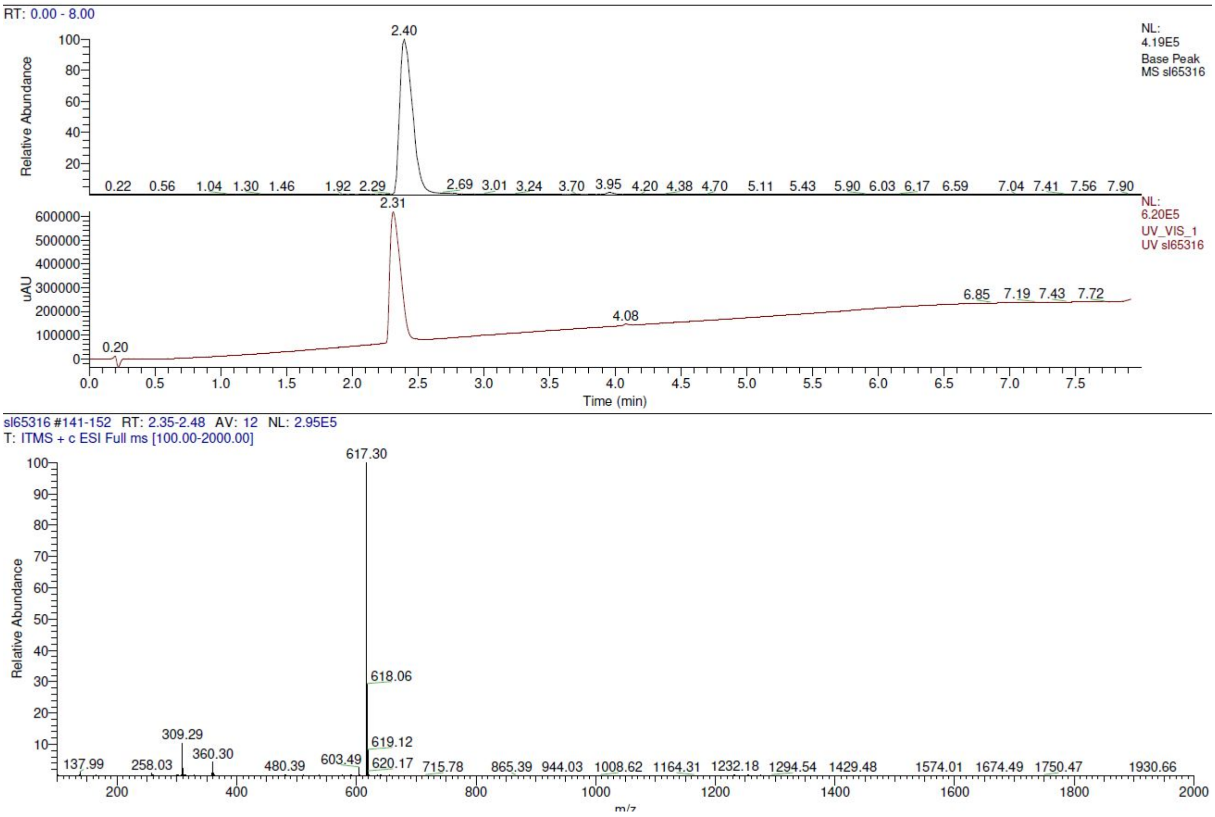

HPLC-MS of L1.

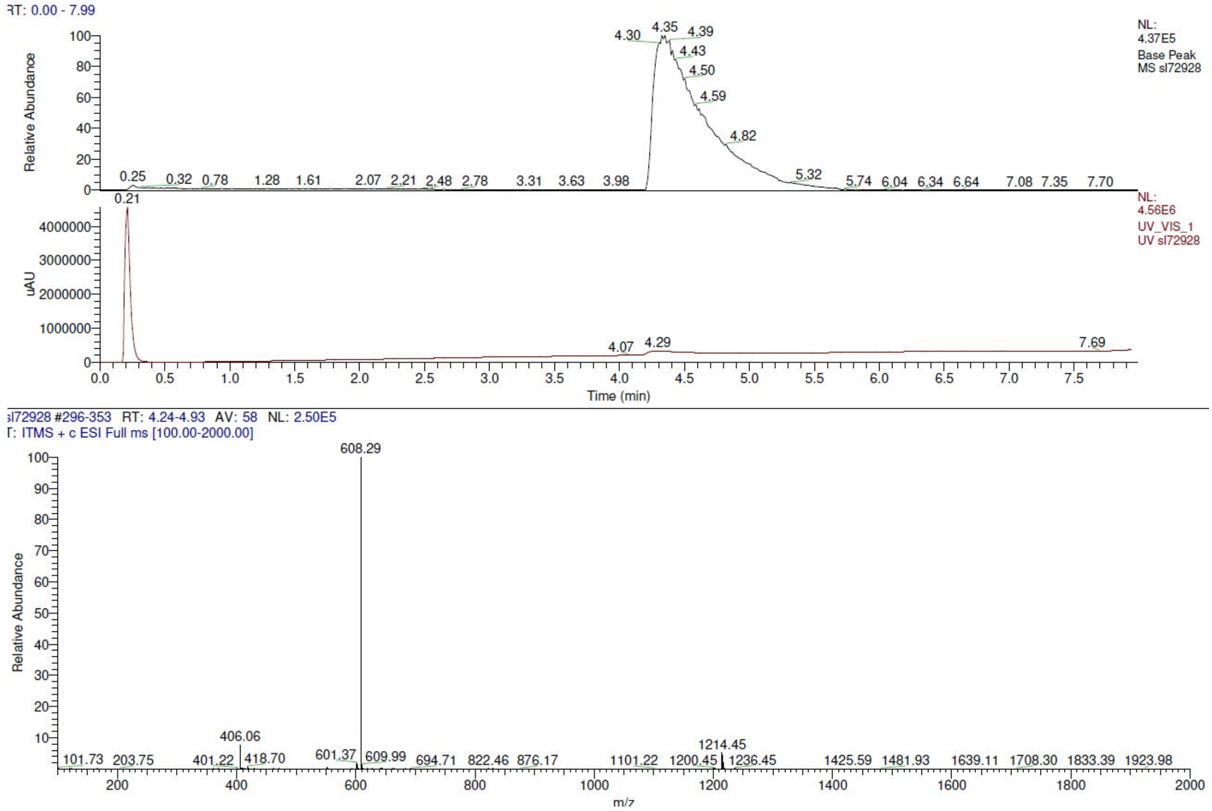

HPLC-MS of L1-Cy5.5.

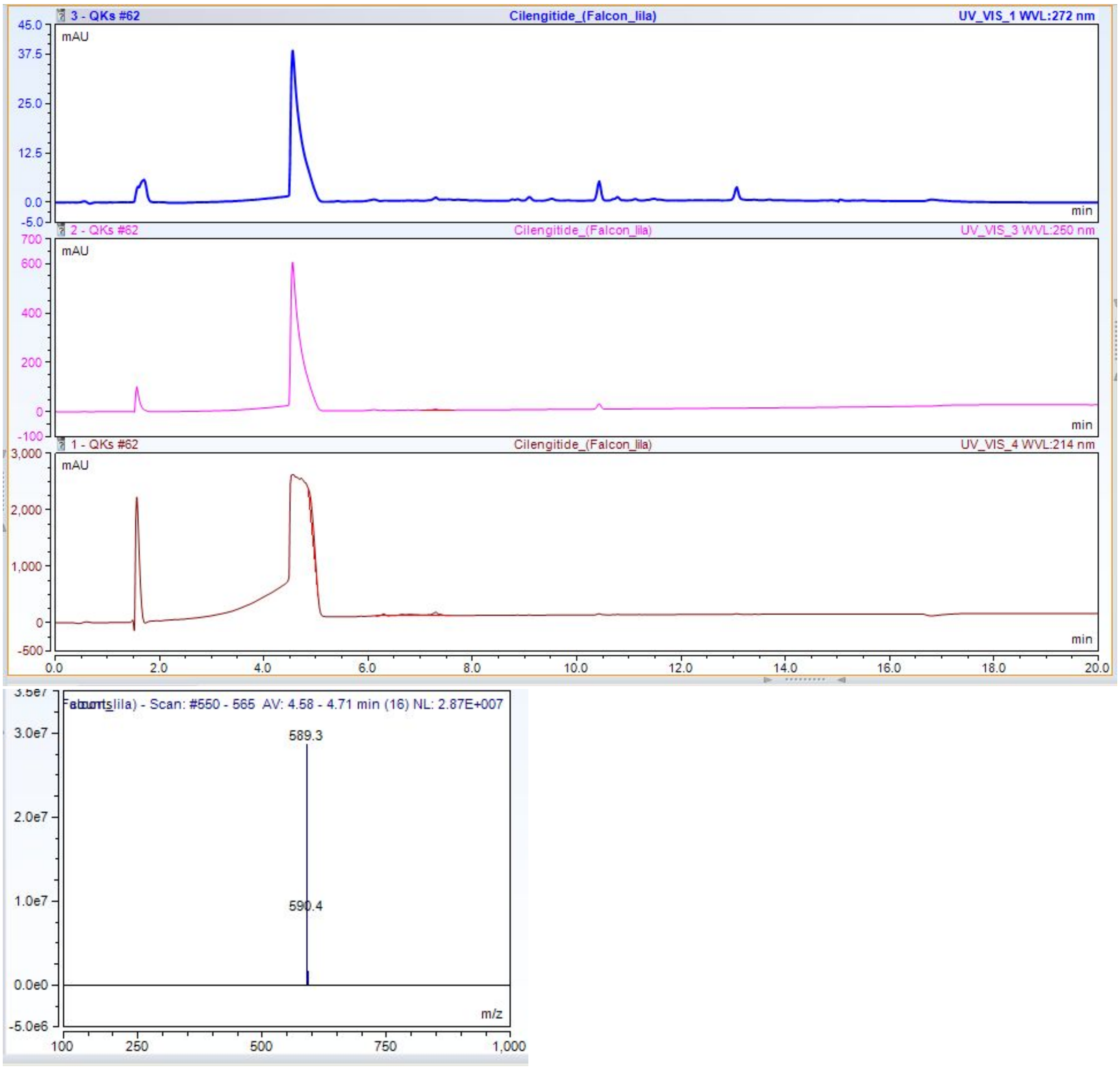

HPLC-MS of Cilengitide (L2).

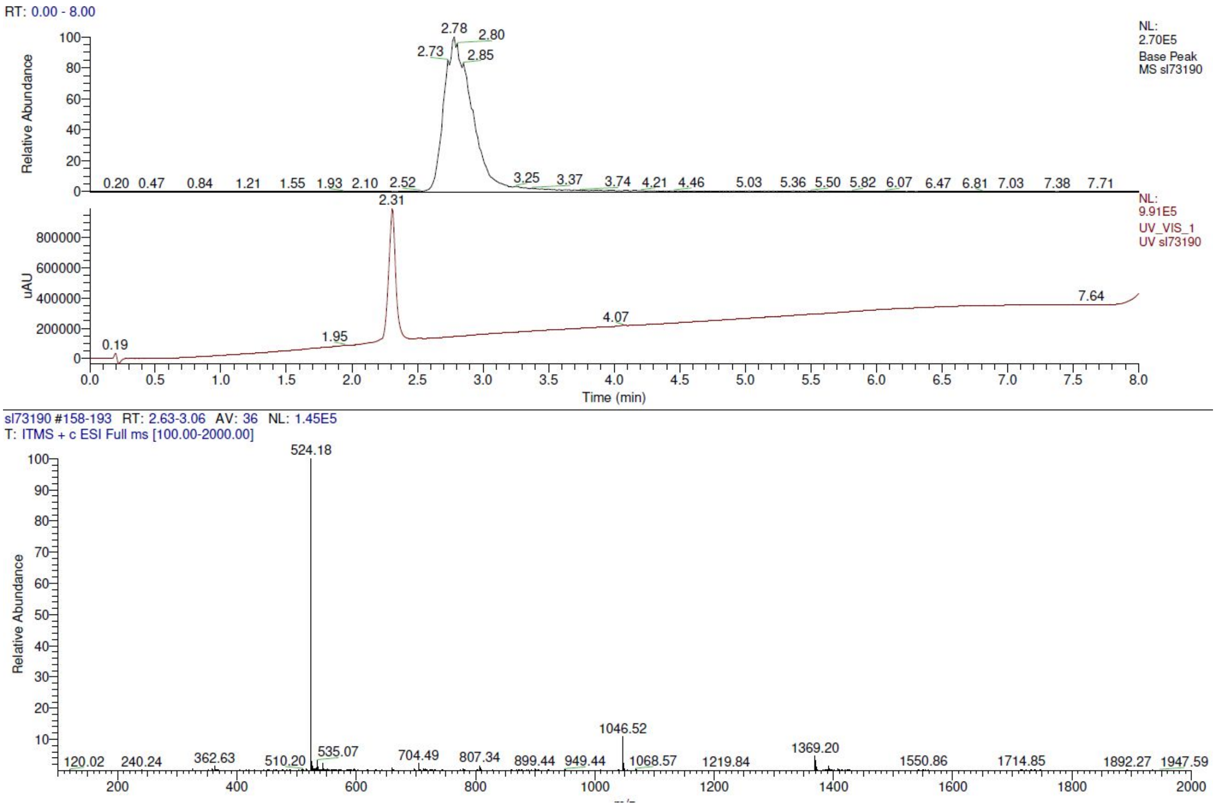

HPLC-MS of L3.

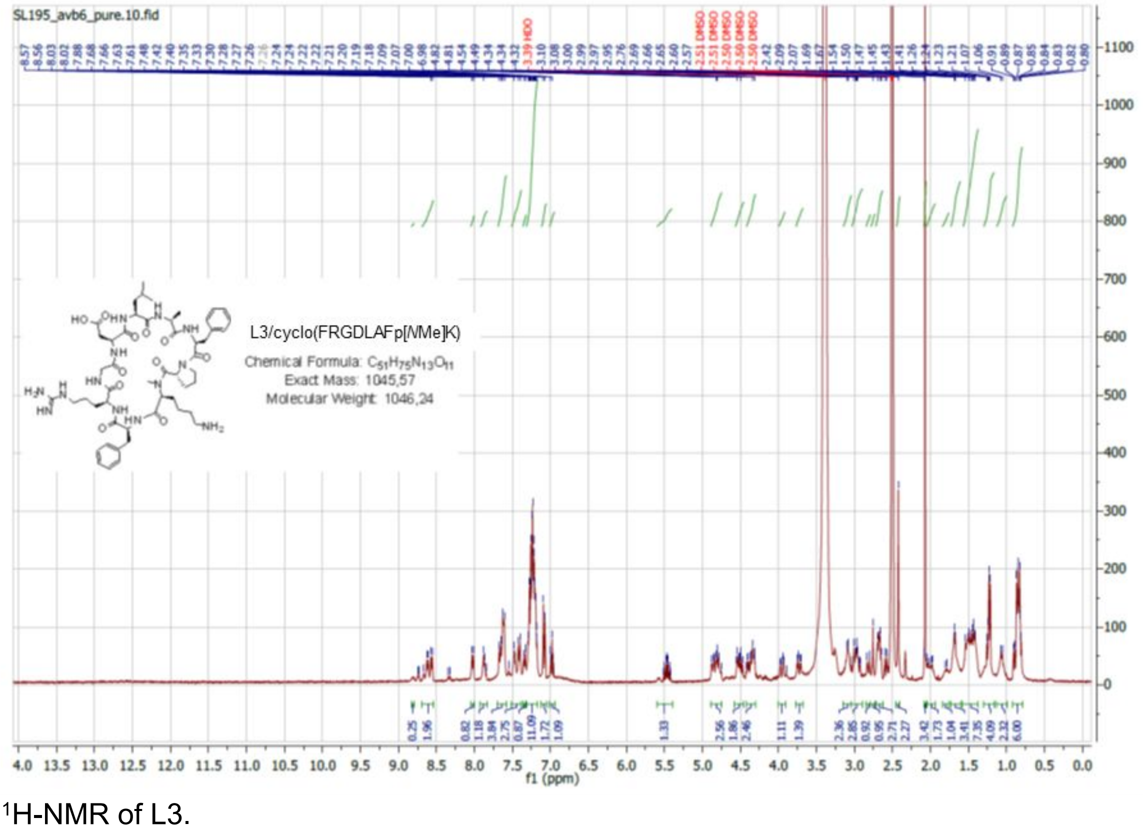

^{1}H-NMR of L3.

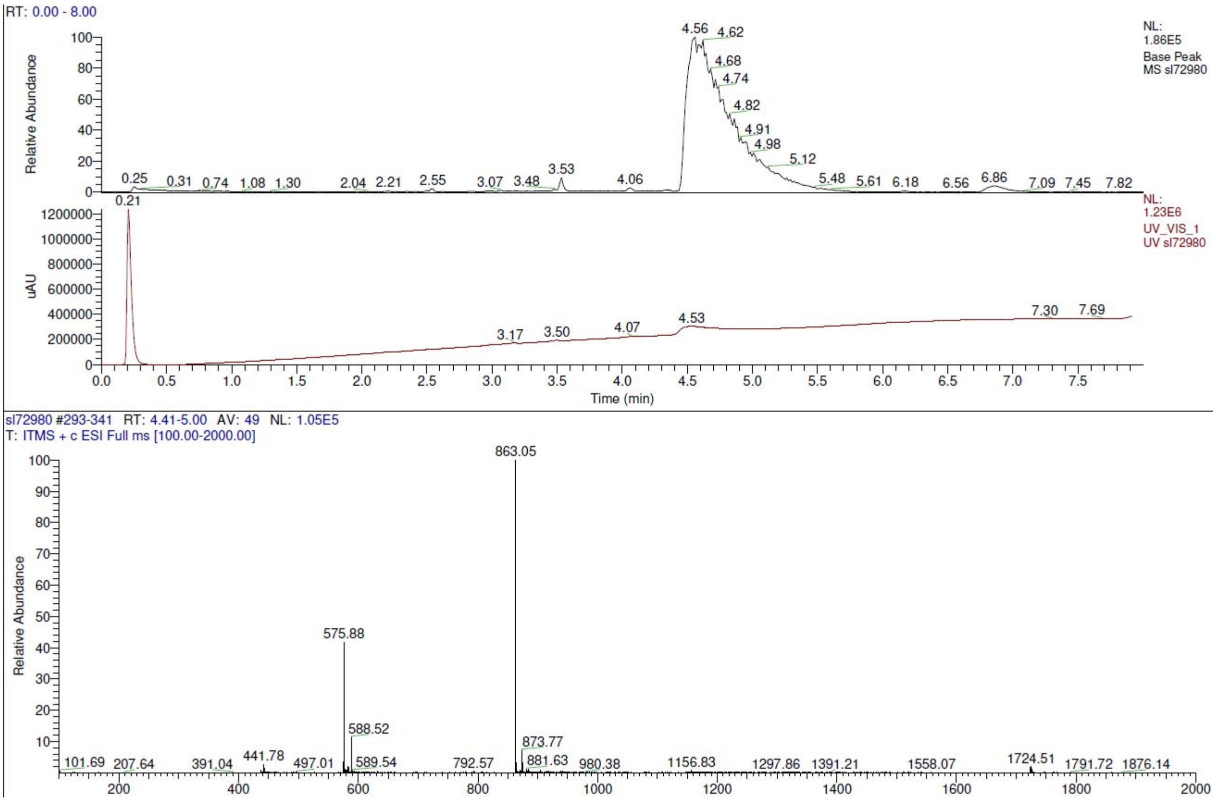

HPLC-MS of L3-Cy5.5.

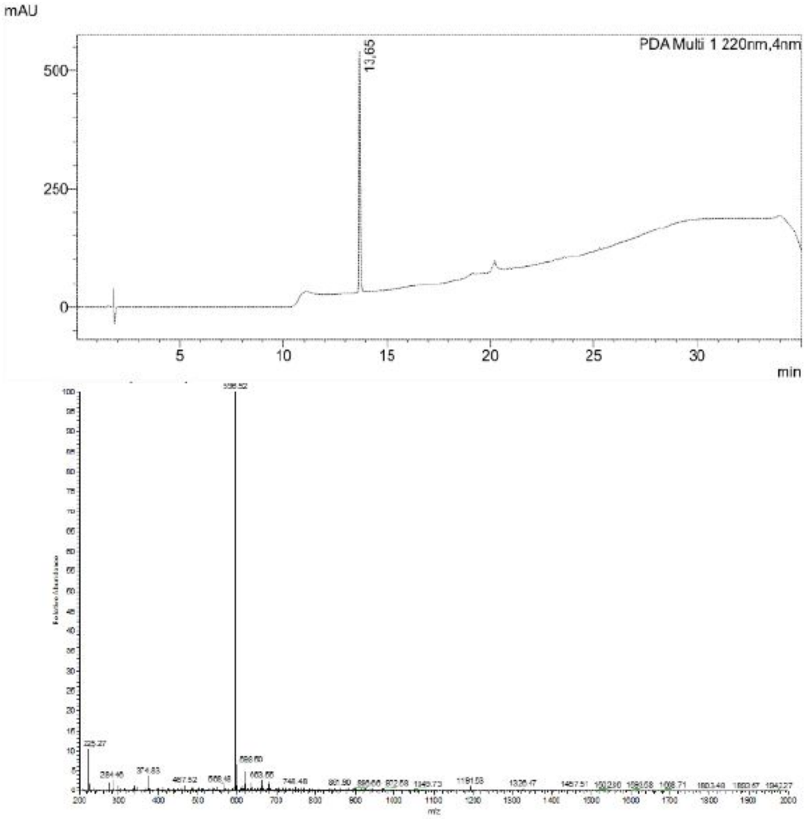

HPLC-MS of L4

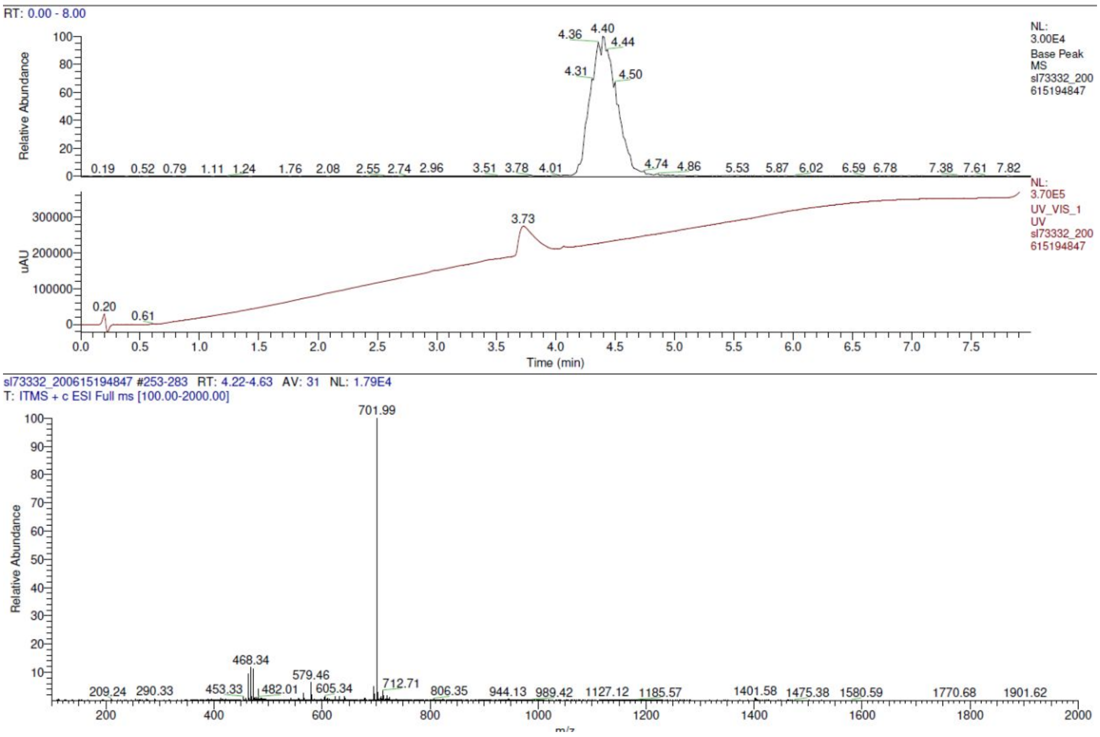

HPLC-MS of L4-Cy5.5.

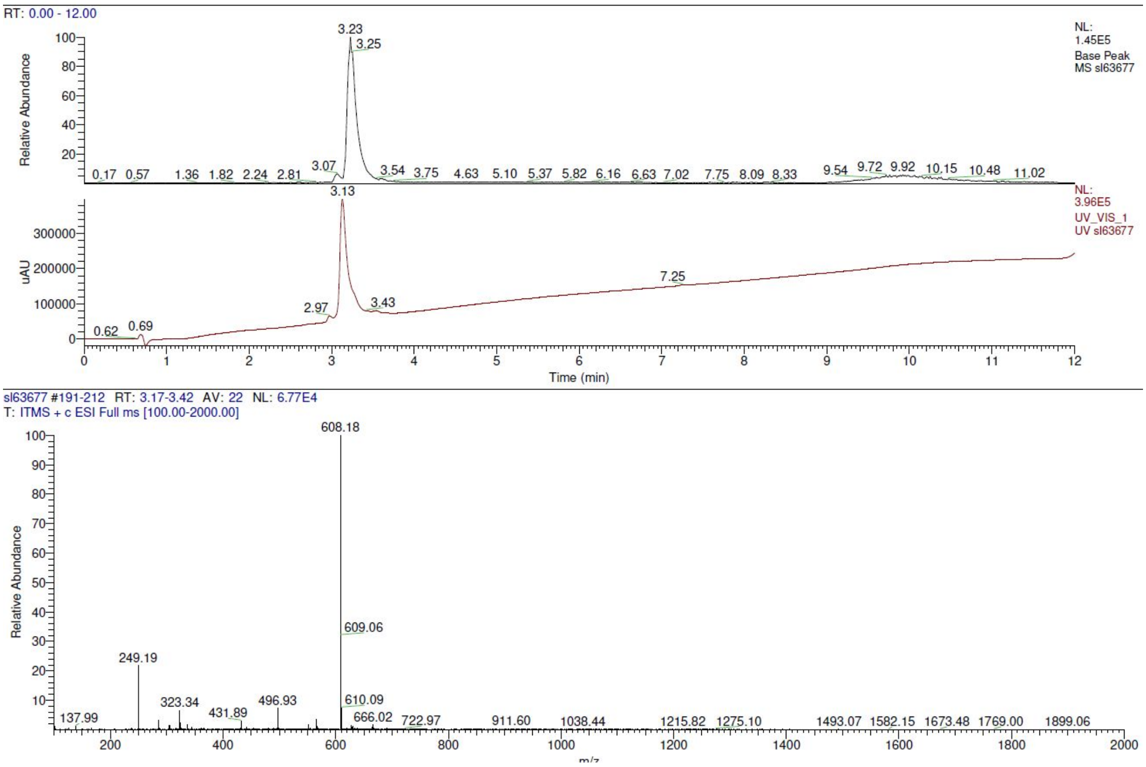

HPLC-MS of L5.

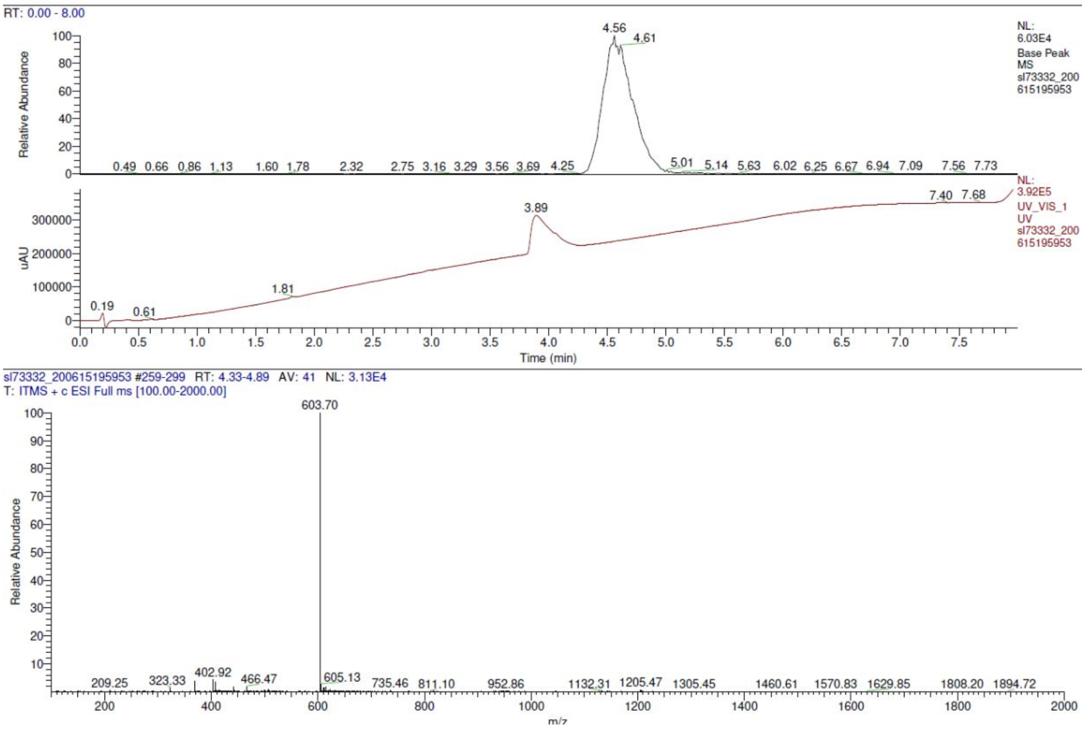

HPLC-MS of L5-Cy5.5.

Scheme S1. HPLC-MS spectrometry was performed using an UltiMate 3000 UHPLC (Dionex) equipped with an LCQ Fleet mass spectrometer (Thermo Scientific) using a long C18 column (Hypersil Gold aQ 175 Å, 3 µm, 150 mm × 2.1 mm) or a short C18 column ("S2", Accucore C18, 80 Å, 2.6 µm, 50 x 2.1 mm) from Thermo Scientific (for Ligands L1, L3, L5, L1-Cy5.5 to L5-Cy5.5). Linear gradients (0.9 mL/min; 8 min or 5 min) of H_2O (0.1% v/v formic acid) and acetonitrile (MeCN; 0.1% v/v formic acid) were used for analytical purpose. Analytical HPLC-MS spectrometry of Cilengitde (L2) was performed on a Vanquish Horizon Flex UHPLC system (Thermo Scientific) using a long C18 column (Hypersil Gold aQ 175 Å, 3 µm, 150 mm × 2.1 mm) from Thermo Scientific. A linear gradient (0.9 mL/min, 15 min) of H_2O (0.1% v/v formic acid) and acetonitrile (MeCN; 0.1% v/v formic acid) was used (10-90% MeCN over 15 min). All final compounds were analyzed via analytical HPLC confirming a purity of ≥95% (220 nm, 250, 272 and/or 214 nm).

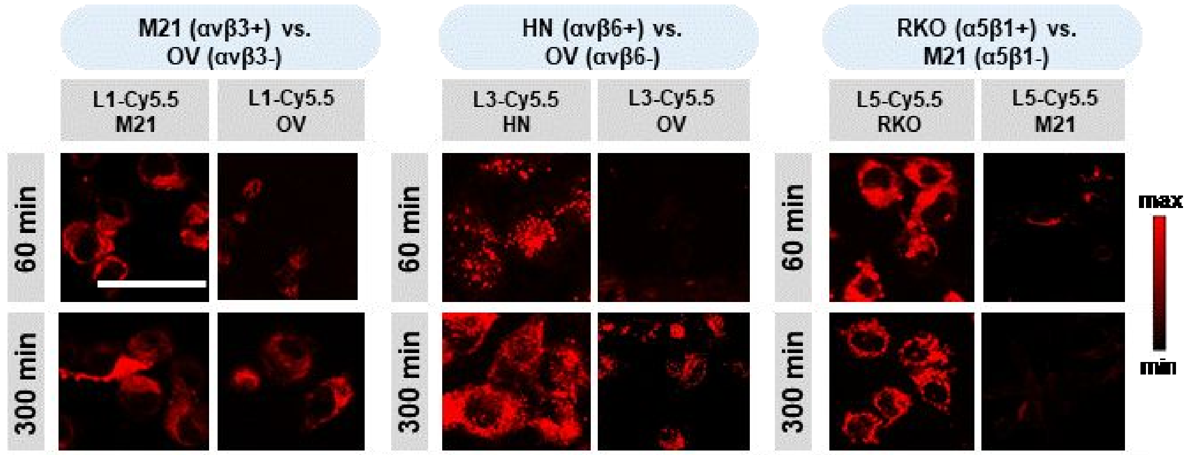

Scheme S2. Controls to integrin-specific cellular uptake of Cy5.5-labeled integrin ligands (to Fig. 7 in the main manuscript). In order to prove integrin-specific cellular uptake, of Cy5.5-labeled integrin ligands, their internalization within 60 or 300 min was compared between cells overexpressing the respective targeted integrin and those with low or lacking expression (scale bar: 100 µm):

- L1-Cy5.5 (αvβ3): M21 (αvβ3+) versus OV-MZ-6 (OV; αvβ3-),
- L3-Cy5.5 (αvβ6): HN (αvβ6+) versus OV-MZ-6 (OV; αvβ6-), and
- L5-Cy5.5 (α5β1): RKO(α5β1+) versus M21 (α5β1-)

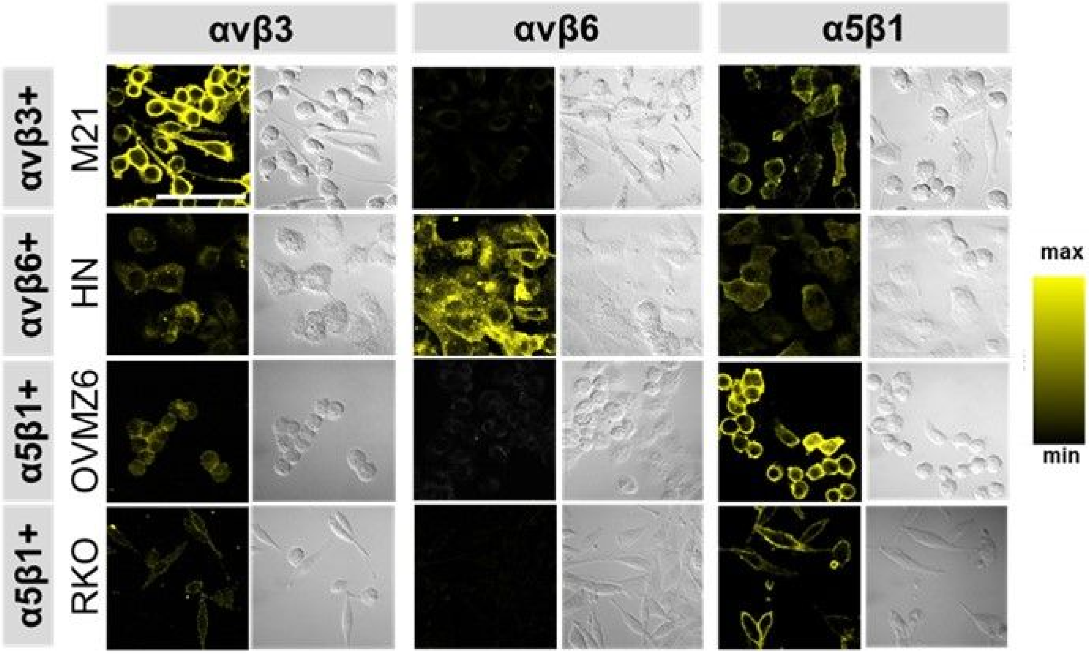

Scheme S3. Identification of cell types overexpressing the respective targeted integrin subtype. Immunostaining of integrins αvβ3, α5β1, and αvβ6, respectively, in αvβ3-overexpressing M21 cells, α5β1-overexpressing OV-MZ-6 and RKO, as well as in αvβ6-overexpressing HN cells was done as described by using primary antibodies raised against αvβ3, α5β1, or αvβ6, followed by a secondary Alexa 568-conjugated goat-anti-mouse or goat-anti-rabbit IgG antibody. Fluorescence images and the corresponding differential interference contrast (DIC) images were obtained by confocal laser-scanning microscopy (CLSM: LSM510, Zeiss, Jena; scale bar: 100 µm).

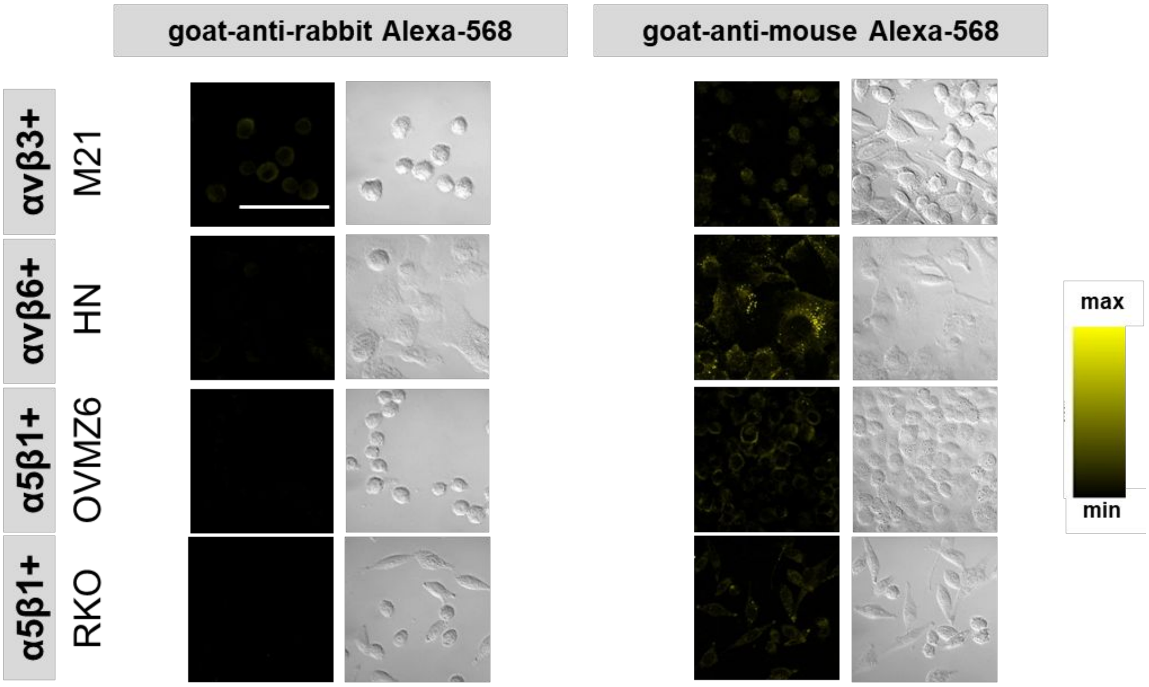

Scheme S4. Controls to the immunostains depicted in Scheme S3 in the absence of the primary integrin-directed antibodies to determine possible unspecific staining of the Alexa-568-labeled secondary antibody. No unspecific behavior of the secondary antibody was detected (λ= 568 nm, scale bar: 100 µM).

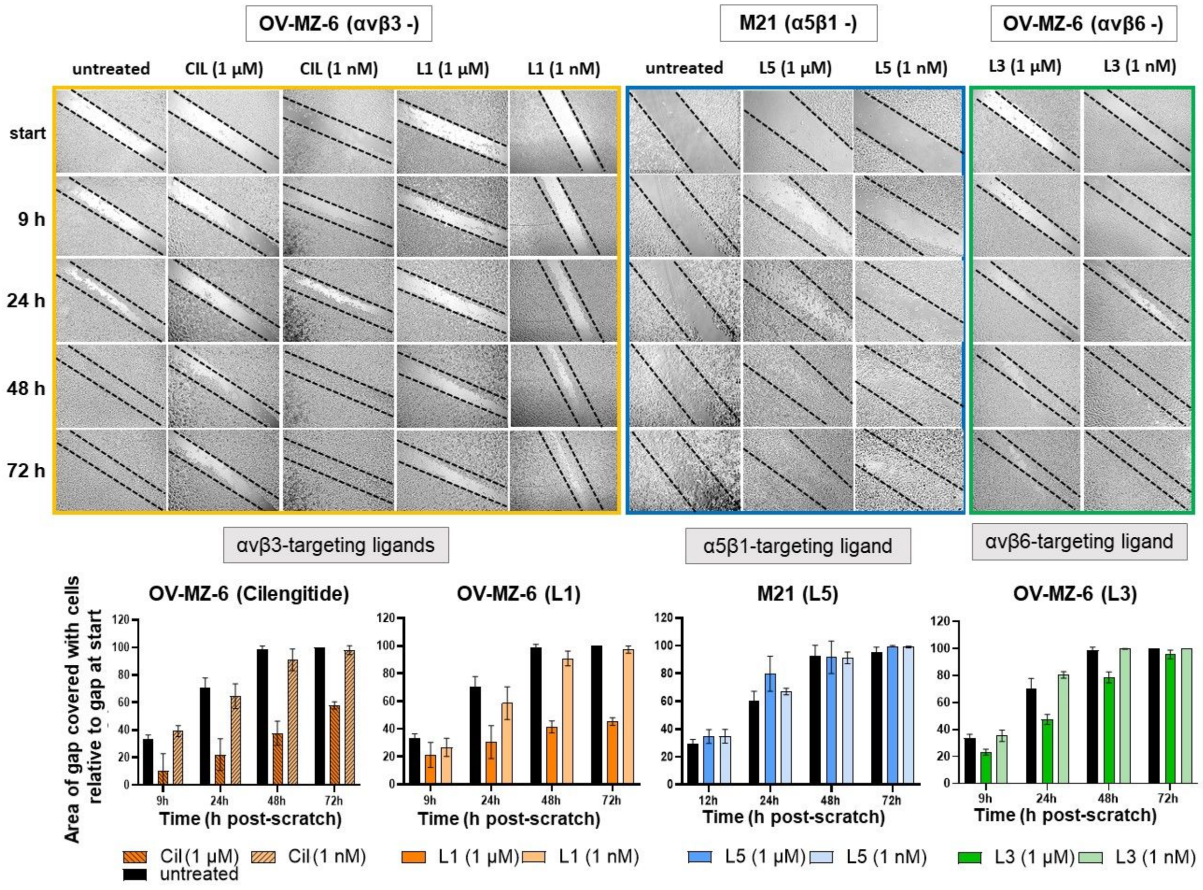

Scheme S5. Effect of integrin ligands on cancer cell migration (control cells to Fig. 10A in the main manuscript). a) Integrin αvβ3- OV-MZ-6 cells, α5β1- M21 cells, α5β1+ and αvβ3- and αvβ6- OV-MZ-6 cells were incubated with 1 µM and 1 nM of Cilengitide (L2; αvβ3), L1 (αvβ3), L5 (α5β1), or L3 (αvβ6) and cell migration monitored by wound scratch assays. Microscopical mages of wound gaps were taken directly after setting the wound scratch and then after 24, 36, and 72 h, respectively. Wound scratch assays were repeated at least 3 times. The extent of wound gap closure [%]/cell coverage was calculated by a python script utilizing Canny's Edge Detection algorithm[11] as described in the *Experimental Section* and summarized in the corresponding histograms.
